# Molecularly Imprinted Solid Phase Extraction Strategy for Quinic Acid

**DOI:** 10.3390/polym14163339

**Published:** 2022-08-16

**Authors:** Sarah H. Megahed, Mohammad Abdel-Halim, Amr Hefnawy, Heba Handoussa, Boris Mizaikoff, Nesrine A. El Gohary

**Affiliations:** 1 Pharmaceutical Chemistry Department, Faculty of Pharmacy and Biotechnology, German University in Cairo, Cairo 11835, Egypt; 2 Division of Molecular Pharmaceutics and Drug Delivery, College of Pharmacy, University of Texas at Austin, Austin, TX 78712, USA; 3 Pharmaceutical Biology Department, Faculty of Pharmacy and Biotechnology, German University in Cairo, Cairo 11835, Egypt; 4 Institute of Analytical and Bioanalytical Chemistry, Ulm University, 89081 Ulm, Germany; 5 Hahn-Schickard, 89077 Ulm, Germany

**Keywords:** computational modelling, molecularly imprinted polymers, solid-phase extraction, quinic acid, *Coffea arabica*

## Abstract

Quinic acid (QA) and its ester conjugates have been subjected to in-depth scientific investigations for their antioxidant properties. In this study, molecularly imprinted polymers (MIPs) were used for selective extraction of quinic acid (QA) from coffee bean extract. Computational modelling was performed to optimize the process of MIP preparation. Three different functional monomers (allylamine, methacrylic acid (MAA) and 4-vinylpyridine (4-VP)) were tested for imprinting. The ratio of each monomer to template chosen was based on the optimum ratio obtained from computational studies. Equilibrium rebinding studies were conducted and MIP C, which was prepared using 4-VP as functional monomer with template to monomer ratio of 1:5, showed better binding performance than the other prepared MIPs. Accordingly, MIP C was chosen to be applied for selective separation of QA using solid-phase extraction. The selectivity of MIP C towards QA was tested versus its analogues found in coffee (caffeic acid and chlorogenic acid). Molecularly imprinted solid-phase extraction (MISPE) using MIP C as sorbent was then applied for selective extraction of QA from aqueous coffee extract. The applied MISPE was able to retrieve 81.918 ± 3.027% of QA with a significant reduction in the amount of other components in the extract.

## 1. Introduction

Oxidative stress is the main cause for altering numerous signaling pathways that eventually promote cellular damage. It is considered a key player mediator in the pathophysiology of several health complications [1]. Intracellular antioxidant enzymes and intake of dietary antioxidants may help maintain the utmost antioxidant balance in the body. Epidemiological studies have proven that the consumption of nutraceuticals with potential antioxidant impacts reduces the risk of several diseases, including neurodegenerative diseases, cardiovascular diseases and cancer, through apoptosis-mediated cytotoxicity. They are also known to reduce inflammation by different mechanisms such as the inhibition of pro-inflammatory transcription factor, nuclear factor Kappa B (NF-κB) [2].

Quinic acid (QA) and its ester conjugates (caffeoylquinic acids) are present in various food products [3] and are the major constituents of coffee [4]. Many reports support the efficacy of nutritional QA in the enhancement of several biological processes via its paramount antioxidant effects [5]. QA has been previously reported as a potent antioxidant due to its capability to lower the intracellular ROS levels in H_2_O_2_ pre-treated cells and inhibition of lipid peroxidation [6]. QA was also found to protect against oxidative stress by increasing the antioxidant capacity as well as decreasing the levels of MDA and nitrite in an in vitro study by Khorasgani et al. [3]. Furthermore, QA upregulated *daf-16 sod-3* expression and downregulated reactive oxygen species (ROS) levels in a *C.elegans* in vivo model [5].

Noteworthily, the ingested QA is used by the human body as a precursor for the synthesis of many important compounds such as nicotinamide [7], which plays a major role in neuronal development and survival [8]. Nicotinamide is also used in the synthesis of two important co-enzymes, nicotinamide adenine dinucleotide (NAD) and nicotinamide adenine dinucleotide phosphate (NADP). Both NAD and NADP are essential in many processes in the human body such as DNA repair, energy production and cell death regulation [9].

Several old techniques have been developed to isolate QA from its original natural source, yet, they are still reports with several limitations [10]. Many extraction protocols and chromatographic methods have been developed to optimize the extraction of QA, such as column chromatography [11], alkaline hydrolysis [12] and liquid–liquid extraction while using an amine as an extractant [13]. However, most of the reported methods are solvent- and time-consuming and none of these methods are considered as having high selectivity towards QA.

Molecular imprinting is a rapidly growing technique used to create synthetic receptors with recognition sites that have the ability to bind specifically to a wide variety of molecules ranging from small drug molecules to large peptides or proteins. The molecularly imprinted polymer (MIP) technique can be described by analogy to the “lock and key model” described by Emil Fischer [14]. The synthesized molecularly imprinted polymers (MIPs) have many cavities complementary to their template molecules in shape, size and chemical functionality, causing them to be particularly selective towards those target molecules [15].

In the past few years, several research articles and reviews have been published, showing the current advances and diversity in the synthesis and application of MIPs [16,17,18]. These studies show the increasing importance of the use of MIPs in the field of analytical chemistry and their application in sensors, extraction and chromatography [19].

The use of MIPs as a replacement for biological material in optical [20] and electrochemical [21] biosensors has attracted much attention throughout the years. This is attributed to their superiority over the biological components in terms of cost, stability and reusability [17]. They also offer outstanding recognition ability, selectivity, specificity and robustness [17,22]. Accordingly, molecular imprinting has become one of the most important techniques for fabricating synthetic ligands on sensor surfaces [17,19]. MIPs have been successfully coupled to surface plasmon resonance (SPR) sensors [23], quartz crystal microbalance sensors [24], luminescence probes [25] and electrochemical sensors [26,27]. Molecular-imprinted fluorescent sensors (MIFS) have been used for detection of several organic molecules and metal ions including proteins [28], caffeine [29] and cocaine [30] in addition to silver [31] and aluminum ions [32]. Moreover, MIP-based SPR sensors have been applied for detection of biomarkers [33], biomolecules [34], pesticides [35] and banned additives [36].

The use of MIPs as sorbents has become one of the most commonly used methods for SPE [37]. They have attracted much attention owing to their numerous advantages such as high selectivity, ease of preparation, low cost, reusability and their potential application to a wide range of target molecules [38]. MISPE has been applied for the extraction of different analytes from biological fluids such as blood, urine and bile [39] in addition to environmental samples and plant tissues [18].

Different techniques of molecular imprinting have been reported for the extraction of antioxidants from natural resources [40,41] such as the isolation of oleuropein from olive leaf extracts [42] and the concentration of tannins from Brazilian natural sources [43].

MIPs were also applied on different plant extracts, including coffee, for isolation of QA derivatives [12]. In a recent study by Kanao et al. [44], poly(ethylene glycol) hydrogels prepared by molecular imprinting were used for selective extraction of quinic acid gamma-lactone (QAGL) from coffee. The synthesized MIPs successfully removed QAGL from freshly brewed coffee at high speed with high yield, which resulted in better-tasting coffee. Moreover, the prepared MIPS were highly selective towards QAGL, which prevented non-specific adsorption of other components in coffee.

Bulk imprinting is considered the most widely used method for preparation of MIPs [45]. It is the method of choice for the imprinting of small molecules as it allows fast and reversible adsorption and the release of the template molecule [46]. Bulk imprinting has been successfully used for small molecules comparable to QA such as sinapic acid [47], gallic acid [48], caffeic acid and *p*-hydroxybenzoic acid [49]. However, the preparation of MIP using QA as a template has not been reported before.

In this work, three bulk MIPs were synthesized using three different monomers. Their binding performance and their ability to be used as sorbents for SPE of QA from coffee beans have been examined.

## 2. Materials and Methods

### 2.1. Reagents and Materials

Standard QA (98%) was purchased from Alfa Aesar. Caffeic acid (CA) (98%), chlorogenic acid (CLA) (95%), acetonitrile (CAN) (HPLC grade; ≥9.99%), methanol (99.8%), absolute ethanol (EtOH) (≥99.5%), formic acid (reagent grade; ≥95%), glacial acetic acid (≥99.7%), methacrylic acid (MAA) (stabilized with hydroquinone monomethyl ether; ≥90.0%), 4-vinylpyridine (4-VP) (contains 100 ppm hydroquinone as inhibitor; 95%), ethylene glycol and dimethacrylate (EGDMA) (contains 90–100 ppm hydroquinone monomethyl ether as inhibitor; 98%) were obtained from Sigma Aldrich (Darmstadt, Germany). Ethyl acetate (EtOAc) (99.5%) was purchased from Alfa Chemical (India) and a purelab UHQ (ELGA) water purification system (High Wycombe, Buckinghamshire, UK) was used to obtain ultra-pure water. Empty polypropylene SPE 3 mL tubes with PE frits of 20 µm porosity were obtained from Supelco Inc. (Bellefonte, PA, USA).

Green coffee (*C. arabica* L.) beans were kindly supplied by Misr Coffee (10th of Ramadan Ind. City, Cairo, Egypt Industrial Company). The beans were mechanically ground and milled into size (40 mesh) for extraction and application steps. The obtained granules were completely dried using a hot air oven at a temperature of 38 °C for 2 h.

### 2.2. Computational Modelling: Monomers Molar Ratio Screening

Gaussian 03 package was used to determine the optimum template to monomer molar ratio for bulk polymers. Gaussview 5.0 software (Gaussian Inc., Pittsburgh, USA) was used first to draw 3D structures of the template, QA, monomers, MAA, allylamine and 4-VP, in addition to template-monomer complexes. All the obtained structures were then optimized to the lowest energy conformation using Hartree-Fock theory with the (6–31 G(D)) basis set. Hartree-Fock is an accurate method for large systems, which makes it easier to screen different monomer ratios for specific templates [50,51]. Different template to monomer molar ratios were screened for each of the used monomers and Equation (1) was used to calculate the binding energies of the complexes.

(1) ΔE=E(template–monomer complex)−[E(template)+nE(monomer)]

where Δ*E* refers to the binding energy of the complex and *n* refers to the monomer number in the template–monomer complexes.

The calculations of the binding energies were conducted in the solvent phase (DMSO) using the polarizable continuum model (PCM) to mimic experimental conditions. In this model, the solvent effect is considered during calculations as it affects the stability and the energy of the template–monomer complexes [52], where the solvent is modelled as a polarizable continuum rather than individual molecules [53].

### 2.3. Bulk Polymers Preparation

Different bulk MIPs were prepared via the non-covalent approach, introduced by K. Mosbach et al. [54], using thermal free radical polymerization. The reaction was performed in a glass vial, by dissolving 0.5 mmol of QA in 6 mL of the porogen, DMSO. This was followed by the addition of suitable amount of monomer and the pre-polymerization mixture was stirred at room temperature for 30 min. Afterwards, the cross-linker ethylene glycol dimethacrylate (EGDMA) was added and the solution was left to stir for 5 min. Following which, 75 mg of the free radical initiator was added and the solution was purged with argon for 3 min to remove oxygen and create inert conditions. The glass vial was sealed and left in an oil bath at 60 °C for 24 h to allow polymerization. For each MIP, a non-imprinted polymer (NIP) was prepared using the same procedure without adding the template. The glass vials were then smashed to obtain the bulk polymers, which were then subjected to crushing, grinding and sieving. The fraction with a particle size of 40–100 µm was collected. The full composition of the prepared polymers is described in Table 1.

### 2.4. Morphology Characterization

The surface morphology of the MIPs and their corresponding NIPs was examined using FEI Quanta 650 environmental scanning electron microscope (ESEM) under high vacuum at a high voltage of 10 kV with a spot size of 3.5 and working distance set to around 10 mm. N_2_ adsorption–desorption isotherms were used to analyze the surface area, pore volume and pore size of all polymers at 77 K via a Quantachrome TouchWin v.1.2 instrument (FL, USA). The polymers were first degassed at 150 °C for 24 h to remove the adsorbed gasses and moisture. The specific surface areas were calculated using the Brunauer–Emmett–Teller (BET) method, while the Barrett–Joyner–Halenda (BJH) method was used to calculate the volume and pore size.

### 2.5. Equilibrium Rebinding Studies

The binding studies were conducted at room temperature by modifying the protocol previously described by Saad et al. [45]. Ten mg of the imprinted and non-imprinted polymers were added to 2 mL of 0.1 mM QA solution prepared in water, methanol or ACN: water (4: 1 *v*/*v*). The suspensions were then left to shake at room temperature for 2 h at 200 rpm using a Thermo Scientific^TM^ MaxQ mini 4000 Benchtop Orbital Shaker (Waltham, MA, USA). This was followed by a centrifugation step at 14,000 rpm for 15 min and the supernatants were filtered through 0.22 polytetrafluoroethylene (PTFE) syringe filters. The concentration of the unbound QA was then quantified using UHPLC-MS/MS. The amount of the rebound QA was calculated using Equation (2)

(2) $$B=\frac{\left(C_{i}-C_{f}\right)\times V\times 1000}{W}$$

where *B* is the amount of rebound template in µmol/g polymer, *C_i_* and *C_f_* represent the initial and final concentrations in mM, respectively, *V* is the volume of the solution in ml and *W* is the weight of used polymer in mg.

The imprinting factor was then calculated using Equation (3)

(3) $$IF=\frac{B_{MIP}}{B_{NIP}}$$

where *IF* is the imprinting factor and *B_MIP_* is the amount of template bound in µmol/g of the MIP, while *B_NIP_* is the amount of template bound in µmol/g of the NIP.

### 2.6. Adsorption Kinetics

The uptake profiles of MIP C and its corresponding NIP were studied over 2 h. This was achieved by incubating 10 mg of the polymer with 2 mL of 0.1 mM QA in methanol for 5, 15, 30, 60 and 120 min. This was followed by a centrifugation step and the supernatants were analyzed using UHPLC-MS/MS and the amount of bound template was determined using Equation (2).

The obtained data were further analyzed to determine adsorption kinetics. The pseudo-first order and pseudo-second order kinetics were used to investigate the mechanism of adsorption of MIP C. The pseudo-first order rate can be expressed in Equation (4)

(4) $$ln\left(q_{e}-q_{t}\right)=lnq_{e}-K_{1}t$$

where *q_e_* and *q_t_* are the binding capacities at equilibrium and at time *t* (µmol/g), respectively, *K*_1_ is the rate constant of pseudo-first order in min^−1^ and *t* is time in min [55].

Pseudo-second order is expressed in Equation (5)

(5) $$\frac{1}{q_{t}}=\frac{1}{K_{2}q_{e}^{2}}+\frac{t}{q_{e}}$$

where *K*_2_ is the rate constant of pseudo-second order in g/µmol.min [56].

### 2.7. Binding Isotherm

Ten mg of MIP C and its corresponding NIP were incubated with 2 mL of QA in methanol over the concentration range of (0.01–0.2 mM) for 2 h. The binding isotherms of both polymers were then obtained by plotting the binding capacity (B) versus the initial QA concentration (C_i_). The results were further analyzed using the Freundlich isotherm model [57] expressed by Equation (6).

(6) $$Log\;B=mLog\;C_{f}+Log\alpha$$

where *B* represents the binding capacity in µmol/g, *m* represents the Freundlich constant or heterogenicity factor ranging from 0 to 1, *C_f_* represents the equilibrium concentration in mM and the constant α represents maximum binding capacity in µmol/g [45].

### 2.8. MISPE Procedure Optimization

MIP C and NIP C were used as sorbent materials for offline-mode solid-phase extraction. Forty mg of each polymer was packed into a 3 mL polypropylene SPE cartridge with a 0.22 PTFE frit placed below the polymer and another similar frit placed above the polymer for secure packing. All trials were performed in triplicate and the analytical measurements were obtained using UHPLC-MS/MS.

A systematic one-factor-at-a-time (OFAT) approach was used to investigate different parameters affecting the extraction procedure including loading amount, loading volume, washing solvent and elution volume. Water: acetic acid (9:1 *v*/*v*) was used as the elution solvent in all trials.

### 2.9. UHPLC-MS/MS Measurements

A new UHPLC-MS/MS method was applied for quantification of QA using ferulic acid as an internal standard. A seven-point calibration curve for QA was prepared in methanol over the concentration range 0.001–0.2 mM.

UHPLC-MS/MS measurements were done using ACQUITY Xevo TQD system (Waters), which is composed of ACQUITY UPLC H-Class system and a XevoTQD triple-quadrupole tandem mass spectrometer with an electrospray ionization (ESI) interface (Waters Corp., Milford, MA, USA). The column used for separation was an Aquity UPLC BEH C_18_ (Waters, Wexford, Ireland), with dimensions of 100 mm × 2.1 mm and stationary phase particle size of 1.7 µm. MassLynx 4.1 software (Waters, Milford, MA, USA) was used for system operation and data acquisition. The TargetLynx quantification program was used to process the acquired data (Waters, Milford, MA, USA). A gradient program was used for chromatographic separation using 0.01% formic acid in water (A) and acetonitrile (B) at a flow rate of 0.3 mL/min, injection volume of 10 µL and column temperature of 40 °C. The gradient was run as follows: 0 min, 90% A, 10% B; 0.75 min, 90% A, 10% B; 2.5 min, 1% A, 99% B; 4 min, 1% A, 99% B; 4.5 min, 90% A, 10% B; 6 min, 90% A, 10% B. The desolvation and cone gas flow rate were 800 and 20 L/h, respectively (nitrogen was used in both cases). The collision gas (argon) was applied at a pressure of 3.67 × 10^−3^ mbar approx. The MS parameters were as follows: radio frequency (RF) lens voltage 2.5 V, capillary voltage 4 kV, source temperature 150 °C and desolvation gas temperature 300 °C. Cone voltage was 45 V and 28 V for QA and ferulic acid, respectively. The ESI source was operated in negative mode. Quantification was performed using multiple reaction monitoring (MRM) of the transitions of m/z 191 > 85 with collision energy of 18 V for QA and m/z 192.89 > 133.95 with collision energy of 14 V for ferulic acid. Dwell time was set automatically by MassLynx 4.1 software.

### 2.10. Method Validation

The applied UPLC-MS/MS method was validated according to the ICH guidelines in terms of linearity, limit of detection (LOD), limit of quantification (LOQ), inter- and intra-day precision and accuracy. More details are provided in the supplementary material.

### 2.11. MIP Cartridge Reusability

MIP cartridge reusability was tested over 10 adsorption–desorption cycles, where the SPE cartridge was filled with 40 mg of MIP C. This was followed by a conditioning step using 2 mL of absolute ethanol, then loading with 2 mL of 0.1 mM QA in ethanol, a washing step using 2 mL of acetonitrile and finally an elution step using 2 mL of water: acetic acid (9:1 *v*/*v*). After the elution step, the cartridge was subjected to 5 washing steps; 2 steps of washing using 3 mL of water: acetic acid (9:1 *v*/*v*) each, then once using 3 mL of water and finally 2 washing steps using 3 mL of absolute ethanol each. The elution fractions were analyzed using the validated UHPLC-MS/MS method and QA recovery percentage was calculated after each elution.

### 2.12. Selectivity Study

**Figure 1:**
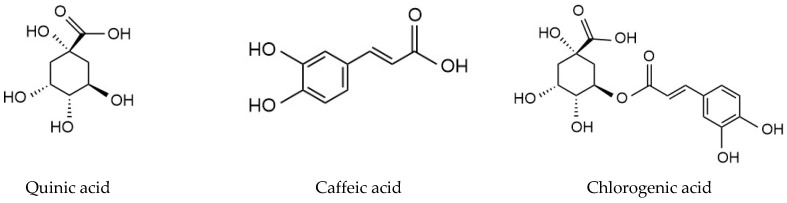
Structures of quinic acid, caffeic acid and chlorogenic acid.

Two mL of equimolar mixture of QA, caffeic acid and chlorogenic acid (Figure 1) (0.05 mM) in ethanol was percolated through SPE cartridges packed with 40 mg of MIP C and NIP C. The cartridges were then washed using 2 mL of acetonitrile. This was followed by the elution step, using 2 mL of 10% acetic acid in UPW.

The obtained elution fraction was evaporated and reconstituted in methanol. The amount of QA in the elution solvent was measured using UHPLC-MS/MS, while caffeic acid and chlorogenic acid were quantified using UHPLC-UV at λ_max_ 325 nm.

### 2.13. MISPE Application on Coffee Extract

#### 2.13.1. UHPLC Method for QA Quantification in Coffee Extract

UHPLC-PDA-ESI- MS and MS/MS analyses were done using the ACQUITY Xevo TQD system (Waters), which is composed of the ACQUITY UPLC H-Class system and a XevoTQD triple-quadrupole tandem mass spectrometer with an electrospray ionization (ESI) interface (Waters Corp., Milford, MA, USA). The column used for separation was an Aquity UPLC BEH C_18_ (Waters, Wexford, Ireland), with dimensions of 100 mm × 2.1 mm and stationary phase particle size of 1.7 µm. MassLynx 4.1 software (Waters, Milford, MA, USA) was used for system operation and data acquisition. The solvent system consisted of 0.01% formic acid in water (A) and acetonitrile (B) by applying the following gradient program: 0 min, 8% B; 30 min, 45% B; 31 min, 8% B; and 33 min, 8% B. The flow rate was 0.2 mL/min and the injection volume was 10 µL. The samples were dissolved in ethanol then filtered through a filter of pore size 0.2 µm. The eluted compounds were detected at mass ranges from 100 to 1000 m/z. The MS scan was carried out at the following conditions: capillary voltage, 3.5 kV; detection at cone voltages, (20 V–95 V); radio frequency (RF) lens voltage, 2.5 V; source temperature, 150 °C and desolvation gas temperature 500 °C. The desolvation and cone gas flow rate were 800 and 20 L/h, respectively (nitrogen was used in both cases). QA was detected through the MRM of the transition m/z 191 > 85 with collision energy of 18 V and cone voltage of 45 V.

#### 2.13.2. Method Validation

The method was validated according to the ICH guidelines in terms of linearity, limit of detection (LOD), limit of quantification (LOQ), inter- and intra-day precision and accuracy. More details are found in the supplementary data.

#### 2.13.3. Preparation of Aqueous Coffee Extract

Fifty grams of roasted coffee beans were subjected to fine grinding and placed in a conical flask, then 1 L of ultrapure water was added. The mixture was heated at 60 °C for 1 h and was left to macerate overnight. This was followed by a centrifugation and a filtration step. Then, the supernatant was concentrated using a rotary vacuum evaporator at 40 °C. The dried residue was stored in an opaque glass bottle for further studies.

#### 2.13.4. Application of MISPE for Extraction of QA from Total Aqueous Coffee Extract

The optimized SPE method was used for the extraction of QA from total aqueous coffee extract. The extract was reconstituted in ethanol: water (97:3 *v*/*v*) and 2 concentrations were prepared, 0.25 mg/mL and 0.5 mg/mL. Two ml of each concentration was loaded to SPE cartridge containing 40 mg of MIP C. This was followed by a washing step using 2 mL of acetonitrile and an elution step using 2 mL of 10% acetic acid in water.

## 3. Results and Discussion

### 3.1. Computational Modelling: Monomers Molar Ratio Screening

**Figure 2:**
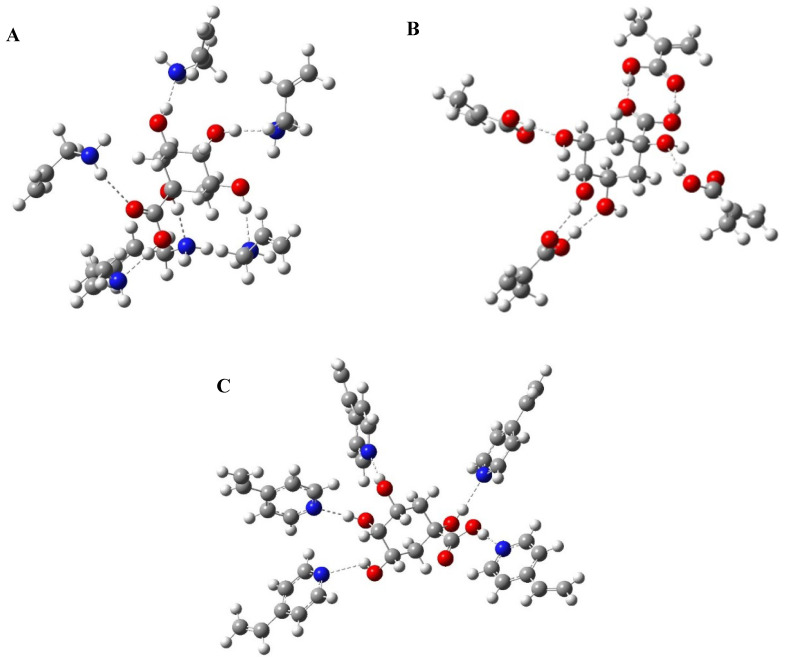
Computer-modelled structures of the best conformations for (**A**) QA–(allylamine)_6_, (**B**) QA–(MAA)_4_ and (**C**) QA–(4-VP)_5_ complexes.

In this study, computational modelling was used to optimize the pre-polymerization complex by determining the most suitable functional monomer molar ratio for each of the chosen monomers, since the self-assembly of the template and functional monomer is the most crucial step in polymer preparation [58]. The study was conducted in the solvent phase and DMSO was the solvent of choice, which was used as the porogen during polymer preparation [15]. The influence of the cross-linker was not considered to simplify the calculations [59]. The three functional monomers used in this study were allylamine, MAA and 4-VP. For each of the chosen monomers, different template: functional monomer ratios were examined. For allylamine, the studied template: monomer ratios were (1:1, 1:2, 1:3, 1:4, 1:5 and 1:6), for MAA, the ratios were (1:1, 1:2, 1:3 and 1:4) and for 4-VP, the studied ratios were (1:1, 1:2, 1:3, 1:4 and 1:5). The optimized structures of QA, functional monomers and pre-polymerization complexes are shown in Figure 2 and Figures S1–S13 in the supplementary data.

Energies of the most stable conformations were then determined and the binding energies of the formed complexes were calculated according to Equation (1) and the results are shown in supplementary data. Based on the binding energies; the best template: monomer ratio was determined for each functional monomer. Results revealed that by increasing the number of monomers used, the calculated binding energies increased, which indicates the formation of more stable complexes [60]. For allylamine, the best ratio was 1:6 (*E* = −175.909 kJ/mol). For MAA, it was 1:4 (*E* = −1633.061 kJ/mol). Finally, for 4-VP the optimum ratio was 1:5 (*E* = −136.5265 kJ/mol) (Figure 2). Accordingly, these ratios were chosen for the synthesis of MIPs and their corresponding NIPs to be used for further applications.

### 3.2. Morphology Characterization

**Figure 3:**
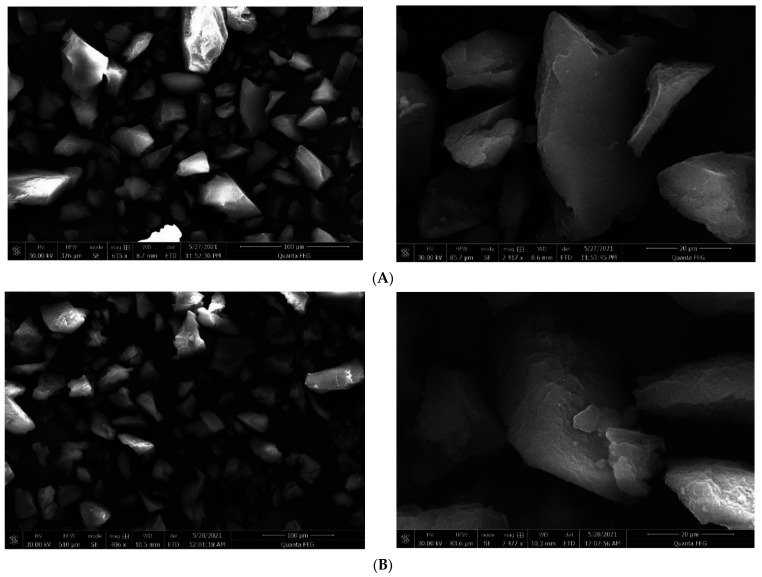
SEM images of (**A**) MIP C and (**B**) NIP C with increasing magnification from left to right.

The surface morphology of MIPs and their corresponding NIPs of particle size range 40–100 µm were analyzed using scanning electron microscopy (SEM) as shown in Figure 3 and Figures S14 and S15 in the supplementary material. The SEM images showed irregular shapes and sizes, which agrees with the nature of bulk MIPs previously reported in literature [45].

**Figure 4:**
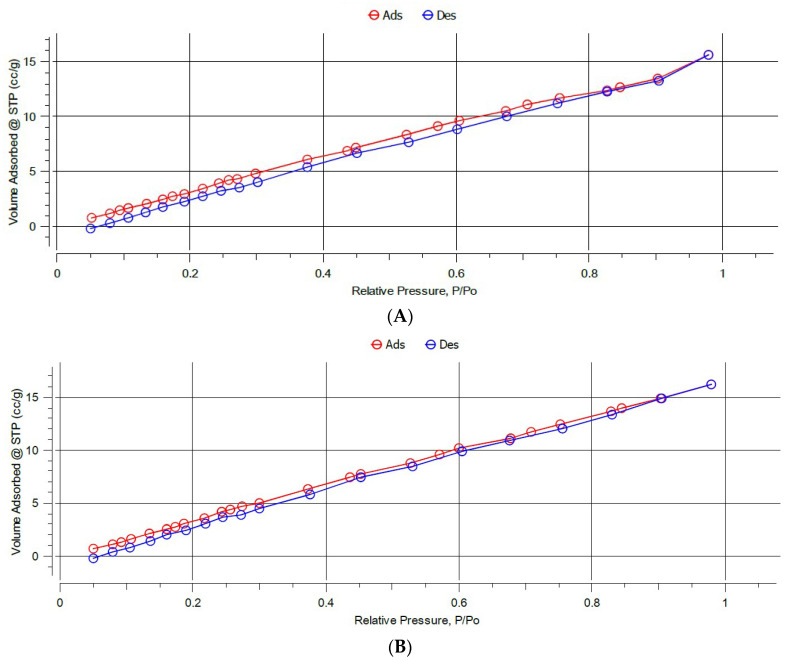
BET isotherms of (**A**) MIP C and (**B**) NIP C.

Nitrogen adsorption–desorption isotherms were performed and BET analysis was used to determine surface areas, while BJH analysis was used to determine the average pore size diameter and pore volume, as these parameters may have a strong impact on the efficiency of adsorption (Figure 4 and Figure S16 in the supplementary material).

The BET results, shown in Table 2 revealed that the MIPs have lower surface areas compared to their corresponding NIPs. This most probably could be attributed to the heterogeneity and roughness of the surface of NIPs which were prepared in the absence of the template, unlike the MIP imprinting process that follows a certain degree of order during the polymerization step [45]. MIP C exhibited the highest surface area (31.41 m^2^/g) compared to MIP A and MIP B that exhibited surface areas of 21.51 m^2^/g and 23.80 m^2^/g, respectively.

The data derived from BJH (Table 2) revealed that all the polymers exhibited a well-developed pore structure. They were all mesoporous with a pore radius of 1.64–1.77 nm, which provides good recognition properties for interaction with the template molecule. These results suggest that the synthesized polymers can be used as sorbents for SPE, since the mesoporous structures are more permeable for solvents compared to micropores and do not require the application of high pressure [61]. All the MIPs and the corresponding NIPs have comparable pore radii, while the pore volumes of all the NIPs are generally larger than the MIPs.

The overall results reveal that all the NIPs showed higher surface areas and porosities compared to the corresponding MIPs. Thus, it may be concluded that the binding performance of the polymers would be attributed to the imprinting process rather than the surface area and porosity of the particles [45].

### 3.3. Rebinding Studies

The synthesized polymers were subjected to batch rebinding studies to evaluate their affinity to QA using 0.1 mM QA solution prepared in three different solvents: water, methanol and acetonitrile: water (4:1 *v*/*v*) as shown in Table 3.

It was observed that when water was used as the rebinding medium, all the polymers showed relatively low binding. This might be attributed to the high solubility of QA in water. This high affinity between QA and water molecules might decrease its interaction with the polymers. Moreover, there was a significant difference between the binding of the MIPs and the corresponding NIPs, pronouncing the specific interaction with the imprinted polymers, where MIP C showed the highest binding capacity of 4.88 ± 0.32 µmol/g, while the binding of its corresponding NIP was 2.28 ± 0.38 µmol/g, with an imprinting factor of 2.14. Although aqueous medium is known to disrupt hydrogen bonding interactions between template and monomer, in the rebinding results of QA a pronounced difference between the binding of QA to the MIPs and the corresponding NIPs was observed. This suggests that hydrogen bonding is not the only factor behind the MIP selectivity towards QA. It can be concluded that, during the imprinting process, different interactions took place based on the size, shape and functionality of the template [62]. During NIP preparation, no proper cavities or recognition sites were formed, therefore the NIP binding to QA was only through non-specific adsorption [63]. As a result, the amount of QA adsorbed by the NIP was lower than by MIP.

It was observed that there was a significant increase in the binding capacity in all polymers when methanol was used as the rebinding medium. This might be because QA has lower solubility in methanol [64], thus a lower affinity to the binding solvent, which increases the chance of interaction between QA and the polymers. It was still observed in this solvent that the binding of QA to MIPs is higher than its binding to the corresponding NIPs, which indicates the success of the imprinting process.

The binding capacities of MIP A, MIP B and MIP C were 7.52 ± 0.64 µmol/g, 3.14 ± 0.36 µmol/g and 9.05 ± 0.80 µmol/g, respectively, while NIP A, NIP B and NIP C showed binding capacities of 4.70 ± 0.28 µmol/g, 1.68 ± 0.32 µmol/g and 5.58 ± 0.53 µmol/g, respectively.

The third binding solvent chosen was ACN, an example of polar aprotic solvent, water was added to acetonitrile with a ratio of ACN:H_2_O (4:1) to ensure the solubility of polar QA, which is insoluble in pure acetonitrile. On comparing the results of rebinding to results obtained in UPW, it was found that the use of acetonitrile increased the interaction between QA and the synthesized polymers, causing an increase in the binding capacity in most of the polymers. It could be argued that the addition of a polar aprotic solvent enhances the hydrogen bond formation between QA and the polymers. Additionally, the low solubility of QA in acetonitrile probably enhanced the interaction between QA and the polymers [65]. It was observed that MIP B and its corresponding NIP prepared using MAA as functional monomer showed lower binding in ACN compared to UPW. This most probably indicates that, in the case of this polymer, the hydrophobic interactions are the main interactions that take place between QA- and MAA-based polymers and this type of interaction is more pronounced when using only UPW as a binding solvent.

MIP C synthesized using 4-VP as functional monomer showed the highest binding capacity in all the rebinding solvents. Although the calculated binding energy of the QA–MAA complex was the highest during computational studies, practically, the polymers prepared with 4-VP showed better overall performance. This can be attributed to the extra interaction between the basic monomer and the acidic template, the pyridine ring of the monomer could promote adsorption because it can form both acid–base interactions and strong hydrogen bonds with the template. Thus, a more stable complex between the template and the functional monomer was formed during the imprinting process [55]. This agrees with what was reported in some studies where 4-VP monomer showed superior results during the imprinting of acidic templates when compared to other acidic or neutral monomers. In a study by Zhang et al., salicylic acid was imprinted using 4-VP and acrylamide as functional monomers, where 4-VP showed superior imprinting effect compared to acrylamide [66]. BET results also revealed that MIP C has the highest surface area (31.41 m^2^/g) when compared to MIP A and MIP B, which might contribute to its higher binding capacity. The binding of the allylamine MIP was higher than the MAA MIP, which also might be attributed to its basic nature.

### 3.4. Adsorption Kinetics

**Figure 5:**
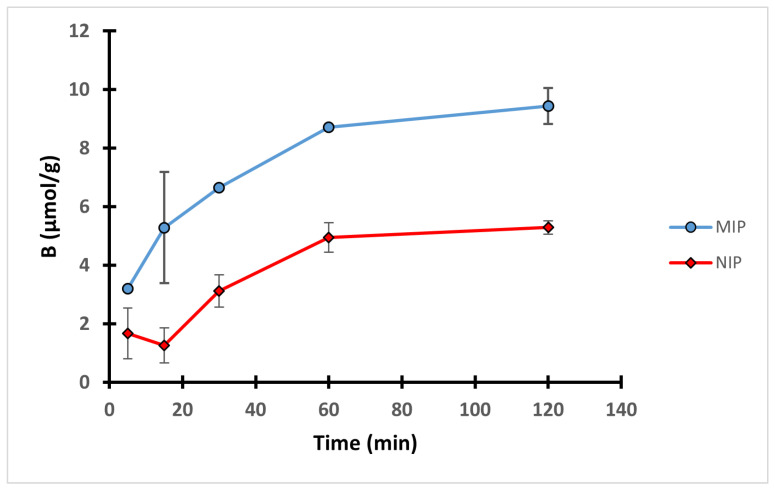
Uptake profiles of MIP C and NIP C over 2 h equilibrium rebinding study in methanol.

In order to study the interactions between the 4-VP polymers and the template during the 2 h equilibrium rebinding study in methanol, an effect of time experiment was conducted on MIP C and NIP C. Ten mg of each polymer was incubated with 0.1 mM QA solution for definite time intervals. The uptake profile shown in Figure 5 revealed that the uptake of both MIP C and NIP C gradually increased during the course of the experiment to reach its maximum after 1 h, after which no significant improvement in the binding performance was observed.

The pseudo-first order and pseudo-second order kinetics were used to investigate the mechanism of adsorption of MIP C (Figure S17 in the supplementary material). The results showed better fitting in pseudo-second order equation where R^2^ was found to be 0.9967 versus R^2^ of 0.8683 obtained from the pseudo-first order equation. This suggests that MIP C binding follows pseudo-second order kinetics with a calculated rate constant of 6.33 × 10^−3^ in g/µmol min.

### 3.5. Binding Isotherm

**Figure 6:**
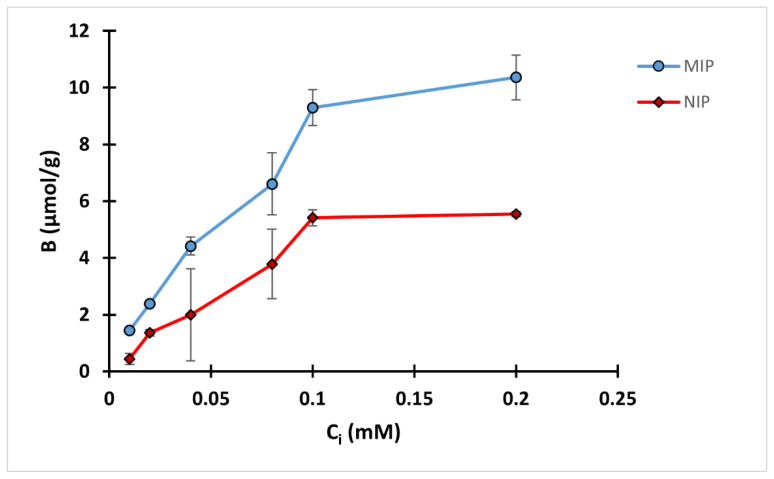
Binding isotherm of MIP C and NIP C.

A two-hour equilibrium rebinding study was carried out by incubating 10 mg polymer with 2 mL QA solution in methanol with different concentrations ranging from 0.01 to 0.2 mM. A binding isotherm was conducted by plotting the binding capacity against initial concentration (C_i_) as in Figure 6. The amount of QA bound increased with the increase of the initial concentration up to 0.1 mM of QA, while there was only a slight difference in the binding capacity between 0.1 and 0.2 mM QA. The results also revealed that the difference between the binding performance of MIP and NIP was more pronounced in the higher concentration ranges, mostly 0.1 and 0.2 mM.

The obtained results were further analyzed using the Freundlich isotherm for MIP C (Figure S18 in the supplementary material). The model was fitted with a high degree of correlation, R^2^ of 0.954. MIP C showed a heterogenicity factor of 0.5389, suggesting heterogenicity of the binding surface, while the α value was 35.3 µmol/g.

### 3.6. MISPE Procedure Optimization

#### 3.6.1. Loading Step Optimization

Different amounts of QA were loaded in SPE cartridges, using methanol as the loading solvent. These amounts were 5, 10, 15 and 20 µmol QA/g polymers. This was followed by a washing step using 2 mL acetonitrile and an elution step using 2 mL of 10% acetic acid in water. The amount of QA in the eluted fraction was calculated and the recovery percent was determined, and the results are shown in Table 4.

The loading amount that showed the highest recovery percent was 5 µmol/g, where the recovery percentage was 72.53 ± 2.68 for the MIP and 56.55 ± 2.77 for the NIP. As the loading amount increased, a decrease in the recovery percentage was attained. This is most probably attributed to occupation of binding sites in the polymer. Therefore, as the loaded amount increased, the fraction of QA that binds to the polymer decreased.

In the following experiment, 5 µmol/g of QA was loaded in different solvents. These solvents were methanol, water, ethanol, ethanol water (1:1 *v*/*v*) and acetonitrile: water (4:1 *v*/*v*). The recovery percent of QA was then calculated and the results are shown in Table 4. It was observed that when water was used as the loading solvent, there was a significant decrease in the recovery % of QA, which agrees with the previously conducted rebinding studies. This confirms that using water decreases the interaction between QA and the polymers.

There was no significant difference between the recovery percent of the MIP and the NIP when using ultrapure water as loading solvent, which is opposite to what was observed during equilibrium rebinding studies. This could be attributed to the short contact time between the template and the polymer in addition to the short length of the polymer column. It could be concluded that increasing the contact time may have a positive impact on the recovery percentage [67].

It was observed that when a mixture of water and an organic solvent was used, (EtOH:H_2_O) or (ACN:H_2_O), only a slight increase in the recovery percent was observed compared to pure water. However, the use of absolute ethanol caused a remarkable increase in the recovery percent, reaching 101.76 ± 1.96.

The corresponding NIP showed a recovery percent of 63.49 ± 5.84. Comparing the two loading solvents, methanol and ethanol, methanol showed lower recovery percent (72.53 ± 1.68), suggesting that the use of less polar organic solvent (with lower dielectric constant, where the dielectric constant for methanol is 32.70 and 24.55 for ethanol) enhances the binding between QA and the MIP. Thus, ethanol was chosen as the loading solvent in further steps.

#### 3.6.2. Washing Step Optimization

The washing step is a crucial step during SPE to maximize the specific interactions between the analyte and the binding sites of the MIP and decrease non-specific interactions [68]. Different washing solvents were used, including acetonitrile, water, ethanol and ethyl acetate. Acetonitrile showed significant increase in the recovery percentage (101.76 ± 1.96) compared to other solvents. It revealed the best selectivity as well as the best retention ability. Acetonitrile is an organic aprotic solvent, where QA is insoluble. This probably enhances the QA–polymer interaction, decreasing the amount of QA lost during the washing step. On the other hand, using water as the washing solvent caused a significant decrease in the recovery percent (44.46 ± 2.55). This could be attributed to the fact that QA is a polar molecule that is highly soluble in water. Additionally, water may disrupt the hydrogen bonds formed between QA and the polymer. For both reasons, some of the QA is lost during the washing step, decreasing its recovery percent. The use of another organic aprotic solvent, ethyl acetate, showed a higher recovery percent (68.38 ± 3.98) compared to water and ethanol, a polar protic solvent. From the obtained results, it was concluded acetonitrile is the optimum washing solvent.

#### 3.6.3. Elution Step Optimization

The desorption of the analyte is achieved using a solvent that develops interactions with the sorbent in order to desorb the analytes retained on the MIP [69]. Water: acetic acid (9:1 *v*/*v*) was chosen as the elution solvent as QA is soluble in water, while the addition of small amount of acetic acid enhanced the disruption of the hydrogen bond between QA and the polymer without causing a major change in the polymer morphology [70].

Different volumes (1 mL, 2 mL, 3 mL and 4 mL) of the elution solvent were used in this step. There was a significant increase in the recovery percentage upon shifting from 1 mL to 2 mL elution solvent. However, on using 3 mL of the elution solvent, there was no significant increase in the recovery percentage, while using 4 mL of the elution solvent decreased the difference in the recovery between the MIP and the NIP. Accordingly, 2 mL of water: acetic acid (9:1 *v*/*v*) was used for elution as it was the lowest amount of solvent to achieve the highest recovery of the analyte.

### 3.7. UHPLC-MS/MS Method Validation

The validated method showed good linearity, LOD, LOQ, precision and accuracy. Further details are provided in the supplementary material.

### 3.8. MIP Cartridge Reusability

**Figure 7:**
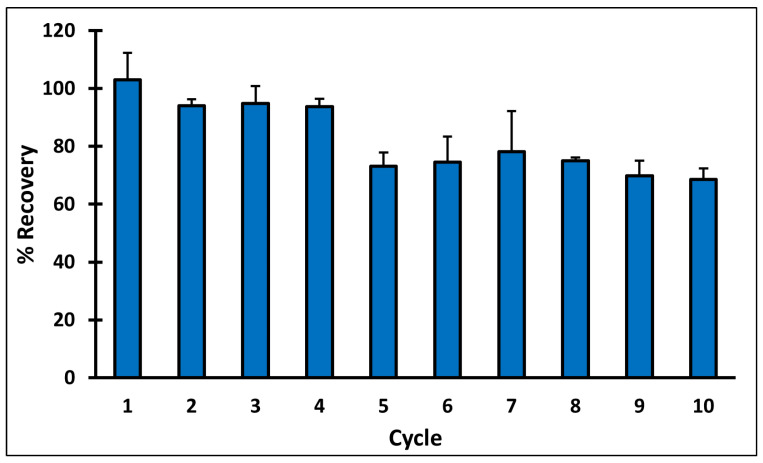
Percent recovery of QA during different cycles of reusing MIP C.

MIP C reusability was studied over ten adsorption–desorption cycles, following the optimized SPE protocol, and QA recovery percentage was calculated after each elution (Figure 7). The results revealed that in cycles 1–4, QA recovery was more than 93%. However, a significant decrease in the recovery was observed in fifth cycle, where the recovery percent reached 73.15 ± 4.77. Cycles 5–8 had comparable results with QA recovery ranging between 73 and 78%, then a small drop was observed in cycles nine and ten, where QA recovery percent reached 69.87± 5.20 and 68.58 ± 3.73, respectively.

### 3.9. MISPE Selectivity

**Figure 8:**
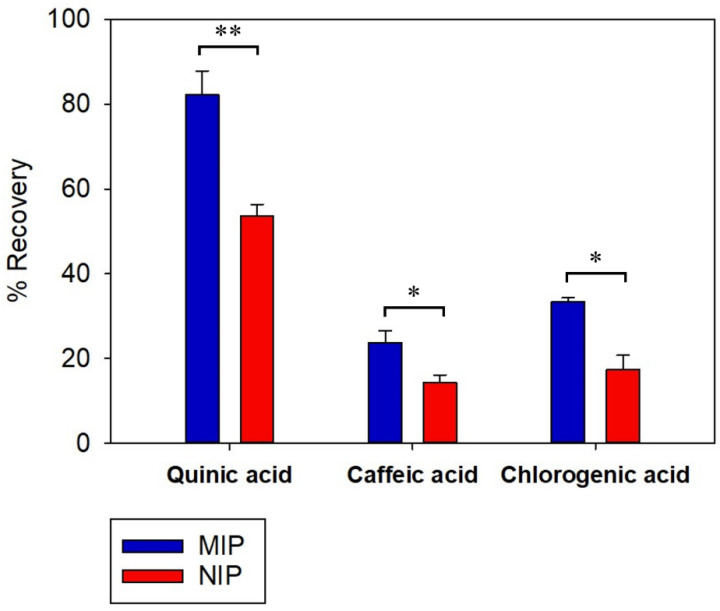
Recovery percentages of QA, CA and CLA upon loading an equimolar mixture of the three compounds to MISPE and NISPE, (*n* = 3), * indicates *p* value ≤ 0.05 and ** indicates *p* value ≤ 0.01.

The selective recognition and retaining properties of the MISPE and NISPE were evaluated. Two compounds were chosen in this study: caffeic acid (CA), having a comparable size to QA, and chlorogenic acid (CLA), an ester of QA and caffeic acid. Both compounds are found in high concentrations in coffee [71]. The results are shown in Figure 8.

Both MISPE and NISPE showed a higher recovery of QA compared to CA and CLA. This difference is probably due to the difference in polarity, where QA is highly hydrophilic with low solubility in acetonitrile, the washing solvent, while CA and CLA exhibit higher solubility in organic solvents. Therefore, some of the CA and CLA may have been removed during the washing step. The recovery percent of QA from MISPE was 82.30 ± 5.58, while it was 53.58 ± 2.77 in case of NISPE. It was observed that for both CA and CLA, the recovery of MISPE was higher than NISPE. For CA, the recovery percent was 23.71 ± 2.85 for MISPE and 14.28 ± 1.84 for NISPE, while for CLA, the recovery percent was 33.41 ± 0.90 for MISPE and 17.46 ± 3.28 for NISPE. This might be due to the structural similarities between QA and the two compounds, where CA is a small molecule with comparable size to QA, it also possesses carboxyl and hydroxyl groups, so it is assumed to bind with some of the functionalities present in the imprinted cavities of the MIP. As for CLA, it has a QA moiety that can fit in the MIP cavities, since CLA is an ester of QA and CA.

### 3.10. MISPE Application on Coffee Extract

#### 3.10.1. UHPLC-MS/MS Method Validation

The validated method showed good linearity, sensitivity, precision and accuracy. Further details are provided in the supplementary material.

#### 3.10.2. MISPE Application on Coffee Extract

**Figure 9:**
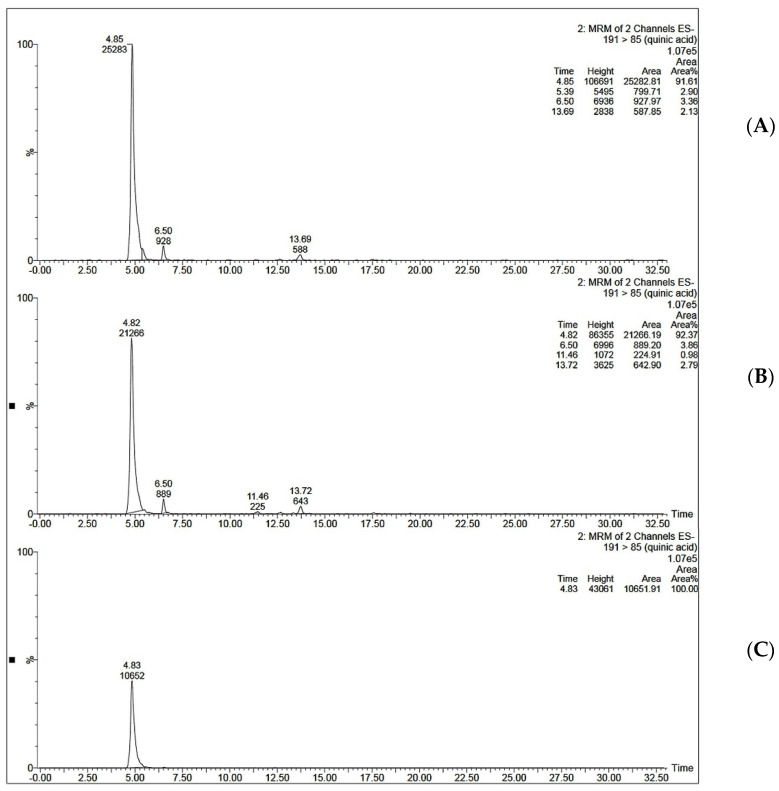
Mass chromatograms of QA (**A**) in 0.25 µg/mL coffee extract dissolved in ethanol: water (97:3 *v*/*v*) before loading to MISPE C, (**B**) the elution fraction obtained from MISPE procedure, (**C**) the elution fraction obtained from NISPE procedure.

**Figure 10:**
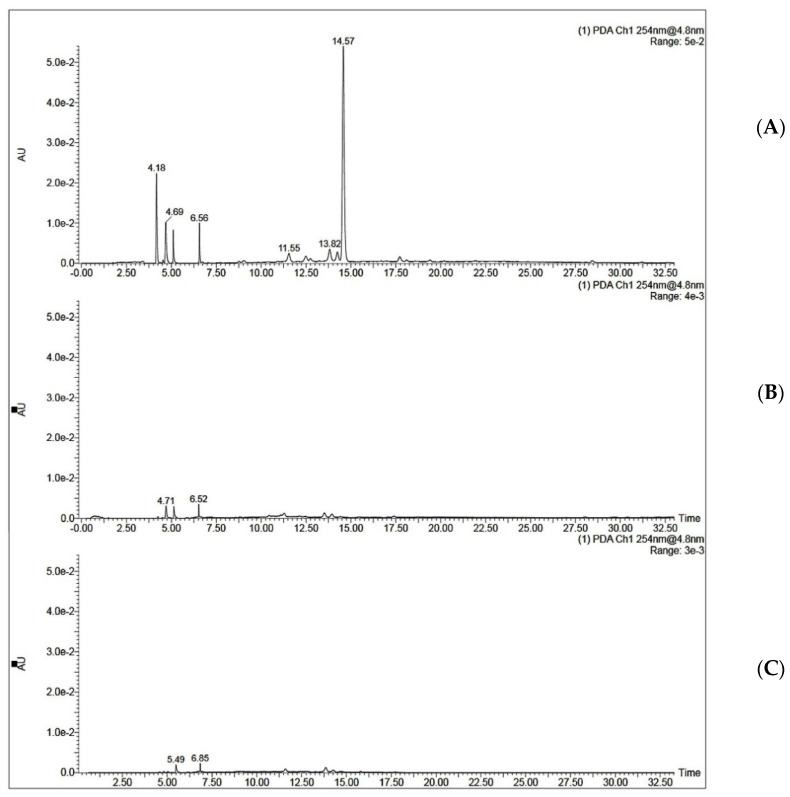
UV chromatograms of QA (**A**) in 0.25 µg/mL coffee extract dissolved in ethanol: water (97:3 *v*/*v*) before loading to MISPE C, (**B**) the elution fraction obtained from MISPE procedure, (**C**) the elution fraction obtained from NISPE procedure.

MIP C was tested for its ability to selectively extract QA from coffee extract. The optimized SPE procedure was applied to the aqueous extract of coffee beans. Two ml of coffee extract (0.25 mg/mL and 0.5 mg/mL) was loaded onto the SPE cartridge. This was followed by a washing step using 2 mL of acetonitrile and elution step using 2 mL of 10% acetic acid in water. It was noticed that when 2 mL of 0.5 mg/mL coffee extract was loaded onto MISPE, the recovery percent was only 36.50 ± 1.19 for MISPE and 28.47 ± 1.22 for NISPE. However, decreasing the concentration of the loaded extract to 0.25 mg/mL showed a significant increase in the recovery percent to reach 81.92 ± 3.03 for MISPE, while the NISPE showed a much lower recovery percent of 37.26 ± 0.84 using the same concentration of the extract (Figure 9). This concludes that the low recovery percent observed while using a higher concentration of the extract could be attributed to the saturation of the binding cavities within the MIP. These results prove that the optimized MISPE is superior to reported conventional methods for QA isolation, such as liquid–liquid extraction previously reported by Tuyun et al. [13], where the maximum QA recovery was found to be 66.906%. The UV chromatogram for aqueous coffee extract before and after loading to MISPE and NISPE shown in Figure 10 revealed that neither the MISPE nor the NISPE were able to bind significantly to any of the other components of the extract, while there was a significant decrease in the amount of the extract components in the elution fractions of both MISPE and NISPE, compared to the original amounts found in the loaded extract.

## 4. Conclusions

The current study represents the use of cheap, selective and simple MISPE procedure for extraction of QA from coffee beans. Three bulk polymers based on three different functional monomers (allylamine, MAA and 4-VP) were synthesized and the molar ratio of each monomer to QA was optimized via computational studies. The 4-VP polymer showed better overall performance in comparison to the other two polymers, thus it was the polymer of choice for SPE application. MIP reusability was tested over ten adsorption–desorption cycles and showed a high recovery of QA (more than 93%) up to the fourth cycle. Selective extraction of QA was observed upon using the optimized MISPE procedure on an equimolar mixture of QA, CA and CGA. The recovery percent of QA was 82.30 ± 5.58, compared to 23.71 ± 2.85 and 33.41 ± 0.90 for CA and CLA, respectively. The application of MISPE for extraction of QA from aqueous coffee extract showed a recovery percent of 81.92 ± 3.03, with a significant reduction in the amounts of other components in the extract. The developed MISPE procedure represents a promising approach for selective extraction of QA from different complex herbal extracts that may be scaled to industrial applications. It can also be applied in the food and beverage industry to decrease the concentration of QA in coffee and enhance its taste. In conclusion, this study succeeded in the isolation of an important nutraceutical in a cost-effective, rapid, robust and reliable method.

## Figures and Tables

**Table 1:** Chemical composition of prepared MIPs.

Polymer	Type of Polymerization	Template (T)	Functional Monomer (FM)	Cross-Linker (CL)	T:FM:CL Molar Ratio
**A**	Bulk	QA	Allylamine	EGDMA	1:6:20
**B**	Bulk	QA	MAA	EGDMA	1:4:20
**C**	Bulk	QA	4-VP	EGDMA	1:5:20

**Table 2:** Surface area, pore volume and pore size of synthesized polymers using BET and BJH methods.

Polymers		A		B		C	
		MIP	NIP	MIP	NIP	MIP	NIP
**BET surface area (m^2^/g)**		21.51	37.13	23.80	39.04	31.41	40.90
**BJH** **Pore volume (cc/g)**	Adsorption	0.02	0.02	0.02	0.02	0.02	0.02
	Desorption	0.02	0.02	0.02	0.02	0.02	0.02
**BJH** **Pore radius (nm)**	Adsorption	1.77	1.64	1.77	1.64	1.77	1.64
	Desorption	1.67	1.67	1.67	1.67	1.67	1.67

**Table 3:** Binding capacities and imprinting factors of bulk polymers in different solvents.

Polymer	FM	Ratio	Water		ACN:Water (4:1)		Methanol	
			B (µmol/g) ± SD	IF	B (µmol/g) ± SD	IF	B (µmol/g) ± SD	IF
MIP A	Allylamine	1:6:20	2.63 ± 0.48	2.48	2.81 ± 0.57	0.82	7.52 ± 0.64	1.60
NIP A	Allylamine	0:6:20	1.06 ± 0.25		3.45 ± 0.49		4.70 ± 0.29	
MIP B	MAA	1:4:20	2.68 ± 0.39	2.15	0.93 ± 0.27	1.69	3.14 ± 0.36	1.87
NIP B	MAA	0:4:20	1.24 ± 0.22		0.55 ± 0.15		1.68 ± 0.32	
MIP C	4-VP	1:5:20	4.88 ± 0.32	2.14	9.97 ± 0.66	1.60	9.05 ± 0.75	1.62
NIP C	4-VP	0:5:20	2.28 ± 0.38		6.25 ± 0.36		5.58 ± 0.53	

**Table 4:** Percent recoveries of QA during SPE optimization.

Trial	Loaded Amount (µmol/g)	Loading Solvent	Washing Solvent	Elution Volume (mL)	MISPE C % Recovery ± SD	NISPE C % Recovery ± SD
**1**	5	MeOH	ACN	2	72.53 ± 1.68	56.55 ± 2.77
**2**	10	MeOH	ACN	2	70.48 ± 2.48	55.01 ± 2.89
**3**	15	MeOH	ACN	2	51.41 ± 2.04	40.59 ± 1.10
**4**	20	MeOH	ACN	2	43.31 ± 2.64	38.62 ± 3.73
**5**	5	H_2_O	ACN	2	50.21 ± 3.24	43.58 ± 4.68
**6**	5	EtOH	ACN	2	101.76 ± 1.96	63.49 ± 5.84
**7**	5	EtOH: H_2_O (1:1)	ACN	2	50.21 ± 2.97	33.87 ± 2.03
**8**	5	ACN: H_2_O (4:1)	ACN	2	54.15 ± 2.03	40.24 ± 1.58
**9**	5	EtOH	H_2_O	2	44.46 ± 6.55	21.57 ± 4.05
**10**	5	EtOH	EtOH	2	56.99 ± 3.28	44.06 ± 5.28
**11**	5	EtOH	EtOAc	2	68.38 ± 3.98	49.24 ± 7.00
**12**	5	EtOH	ACN	1	74.77 ± 3.47	47.19 ± 2.18
**13**	5	EtOH	ACN	3	95.77 ± 5.60	60.76 ± 4.93
**14**	5	EtOH	ACN	4	98.79 ± 3.10	77.10 ± 5.16

## Supplementary Materials

The following supporting information can be downloaded at: https://www.mdpi.com/article/10.3390/polym14163339/s1. Figure S1: Computer modeled structures of the best conformations for (a) QA, (b) 4-VP, (c) allylamine, (d)MAA. Figure S2–S13: Computer modeled structures of the best conformations for QA-FM complexes. Figure S14: SEM images of (a) MIP A and (b) NIP A. Figure S15: SEM images of (a) MIP B and (b) NIP B. Figure S16: BET isotherms of (A) MIP A, (B) NIP A, (C) MIP B and (D) NIP B. Figure S17: (A) Pseudo-first order kinetics and (B) pseudo-second order kinetics for MIP C. Figure S18: Freundlich isotherm for MIP C. Figure S19: Calibration curve of QA in methanol over the concentration range of 0.001–0.2 mM. Figure S20: Calibration curve for QA in ethanol over concentration range of 0.2–40 µg/mL. Table S1: The calculated binding energies for complexes prepared in solvent phase. Table S2: Intra-day and Inter-day precision of QA determination in UPLC-MS/MS method. Table S3: Accuracy of QA determination in UHPLC-MS/MS method. Table S4: %RSD of inter-day and intra-day precision assay for UHPLC measurements. Table S5: Recovery % of spiked QA amount 1x, 2x, and 3x the amount of QA present in coffee extract (10, 20, and 30 µg/mL).
