# Supplementary material for: Molecularly Imprinted Solid Phase Extraction Strategy for Quinic Acid
Source: Polymers (Basel). 2022 Aug 16;14(16):3339. doi: 10.3390/polym14163339 (PMC9416653; doi:10.3390/polym14163339)
Supplement: Supplementary file 1 [file polymers-14-03339-s001.zip › polymers-1823822-supplementary.pdf]

Molecularly Imprinted Solid Phase Extraction Strategy for Quinic Acid Supplementary Data

Sarah H. Megahed [1], Mohammad Abdel-Halim [1], Amr Hefnawy [2], Heba Handoussa [3], Boris Mizaikoff [4,5,*] Nesrine A. El Gohary [1,*]

Computational Modeling: Monomers Molar Ratio Screening

The study was conducted in the solvent phase and DMSO was the solvent of choice which was used as the porogen during polymer preparation. The three functional monomers used in this study were allylamine, MAA and 4-VP. For each of the chosen monomers, different template: functional monomer ratios were tried. For allylamine; the studied template: monomer ratios were (1:1, 1:2, 1:3, 1:4, 1:5 and 1:6), for MAA; the ratios were (1:1, 1:2, 1:3 and 1:4) and for 4-VP; the studied ratios were (1:1, 1:2, 1:3, 1:4 and 1:5). The optimized structures of quinic acid, the functional monomers and the pre-polymerization complexes are shown in **Figures S1 – S13**

Energies of the most stable conformations were then determined and the binding energies of the formed complexes were calculated and the results are shown in **Table S1**. Based on the binding energies, the best template: monomer ratio was determined for each functional monomer. For allylamine; the best ratio was 1:6 (E=-175.909 kJ/mol). For MAA; it was 1:4 (E=-1633.061 kJ/mol). Finally, for 4-VP; the optimum ratio was 1:5 (E=-136.5265 kJ/mol). Accordingly, these ratios were chosen for the synthesis of MIPs and their corresponding NIPs to be used for further applications.

Morphology Characterization:

SEM images of MIP A, MIP B and their corresponding NIPs are shown in **Figures S14 and S15**.

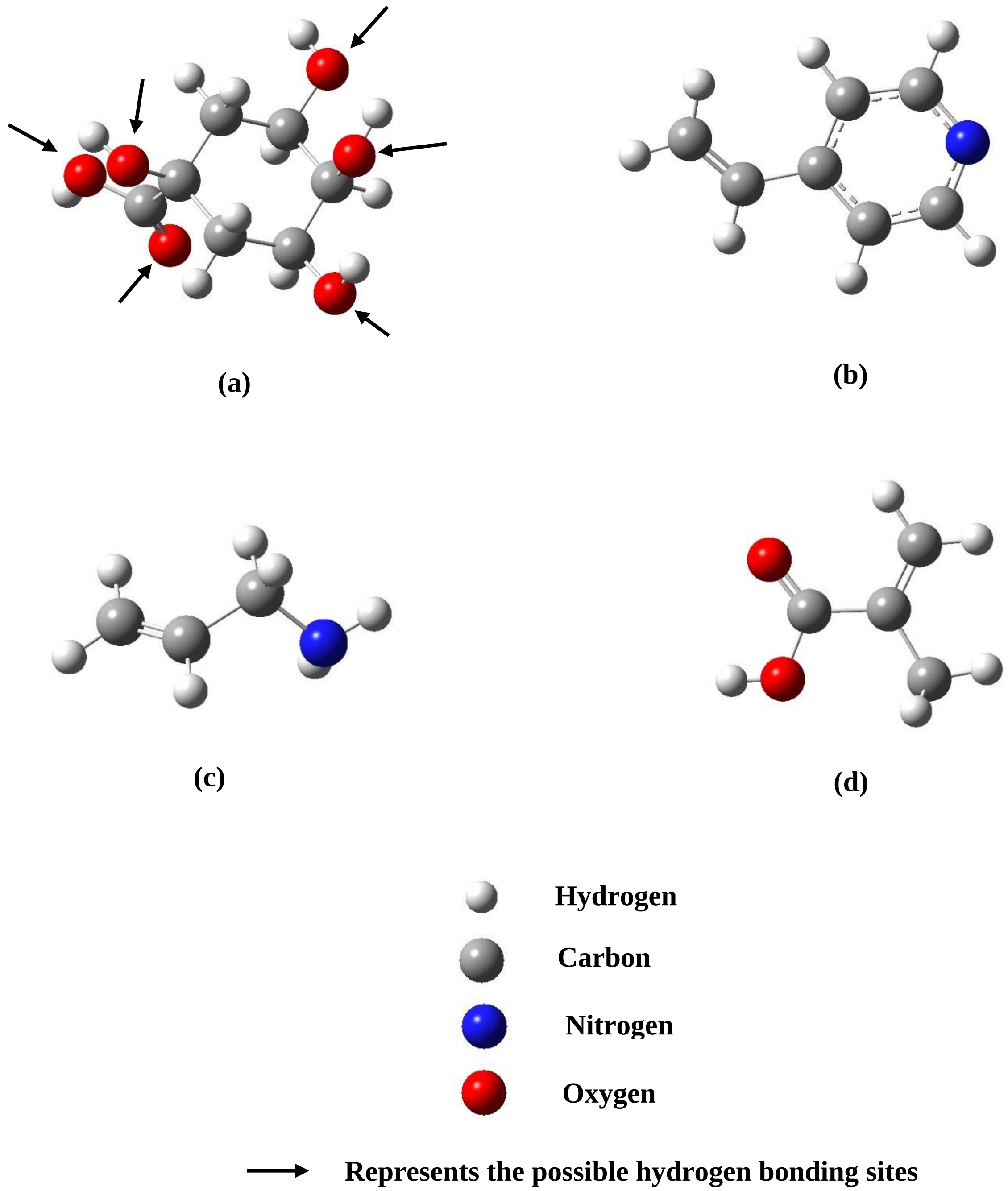

Figure S1: Computer modeled structures of the best conformations for (a) quinic acid, (b) 4-VP, (c) allylamine, (d)MAA.

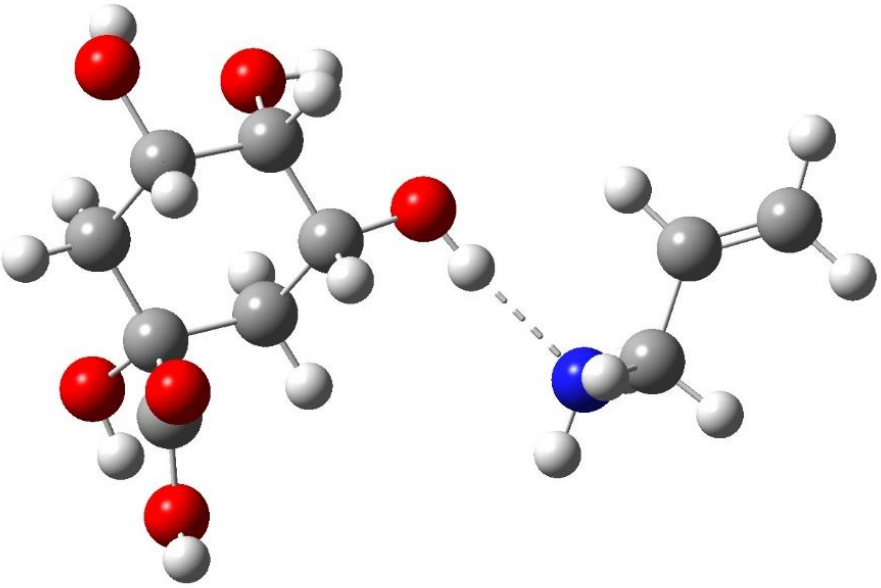

Figure S2: Computer modeled structure of the best QA-(allylamine) complex

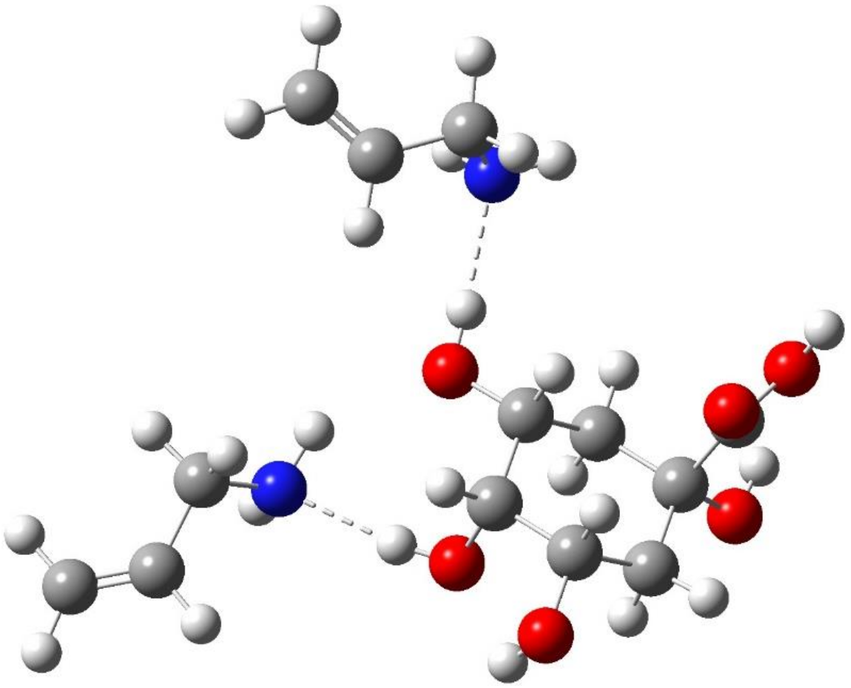

Figure S3: Computer modeled structure of the best QA-$(allylamine)_2$ complex.

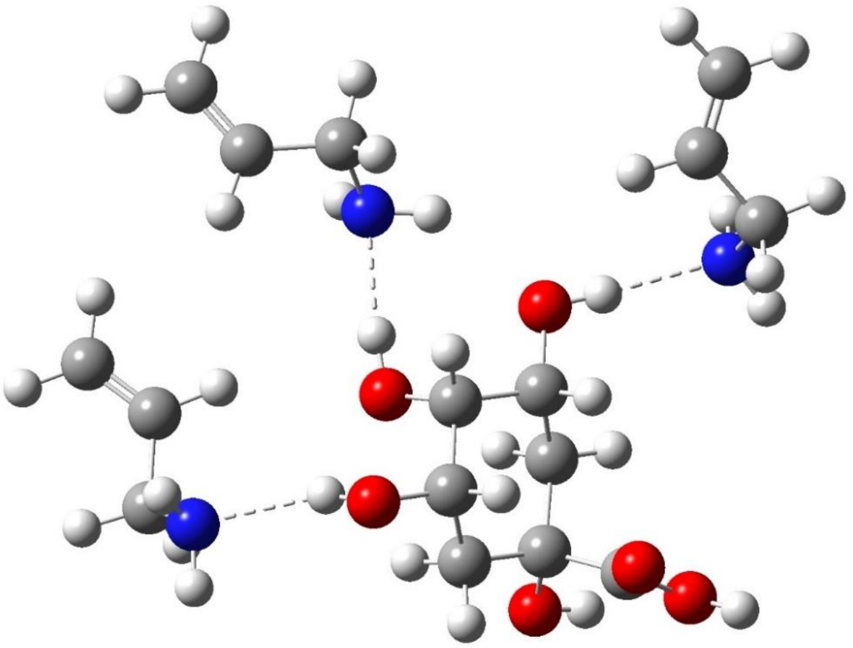

Figure S4: Computer modeled structure of the best QA-(allylamine)$_3$ complex.

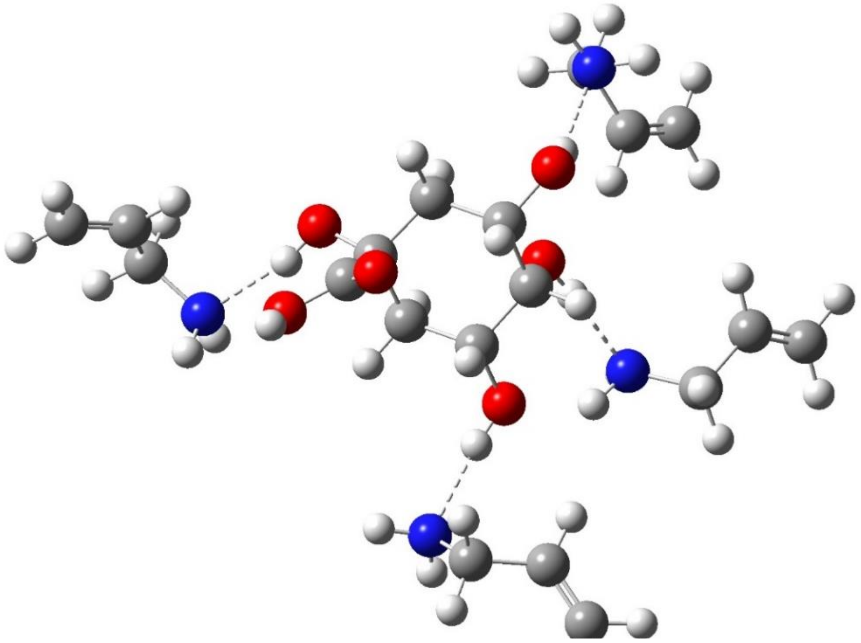

Figure S5: Computer modeled structure of the best QA-(allylamine)$_4$ complex.

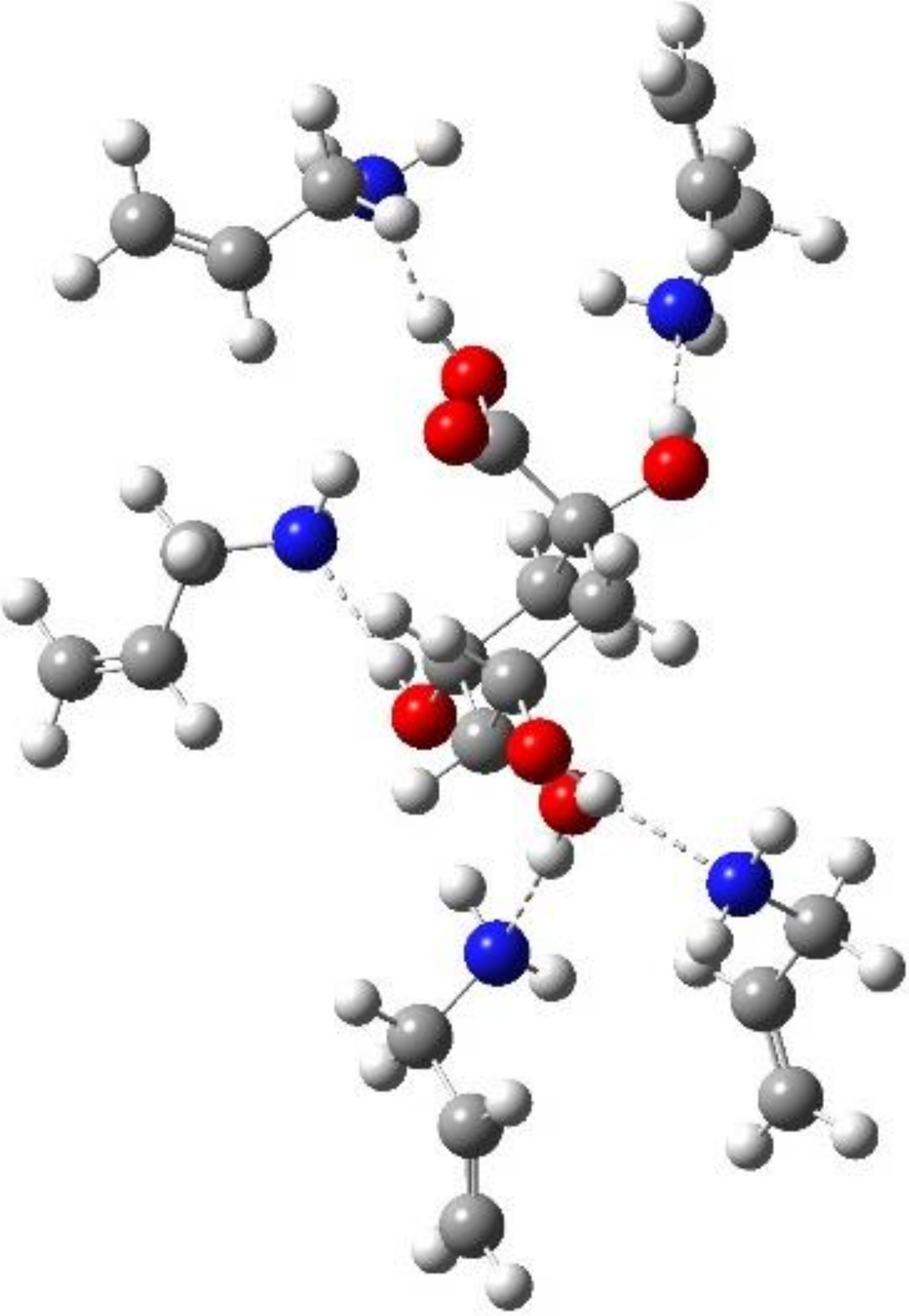

Figure S6: Computer modeled structure of the best QA-(allylamine)$_5$ complex.

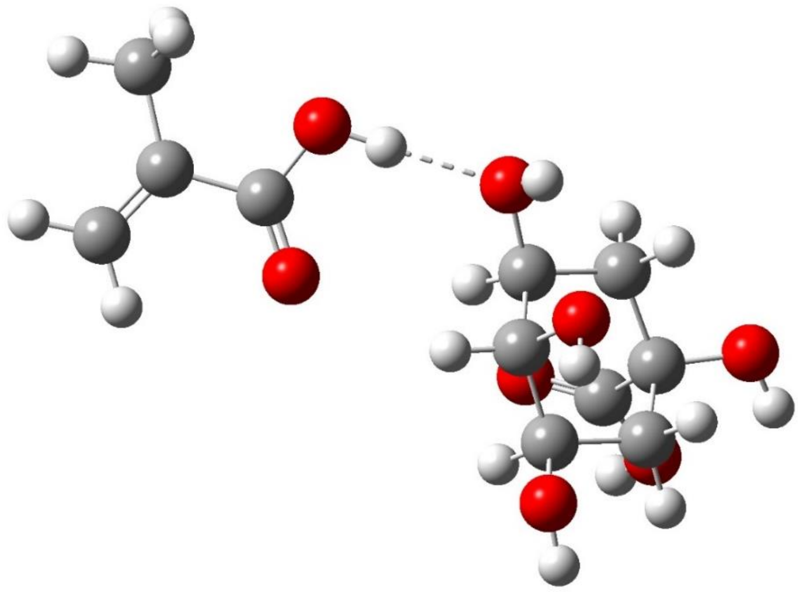

Figure S7: Computer modeled structure of the best QA-(MAA) complex.

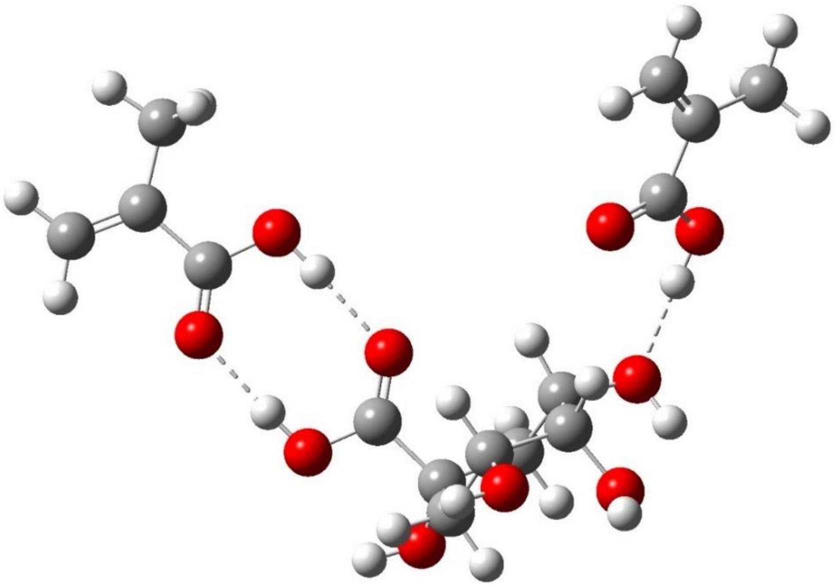

Figure S8: Computer modeled structure of the best QA-$(MAA)_2$ complex.

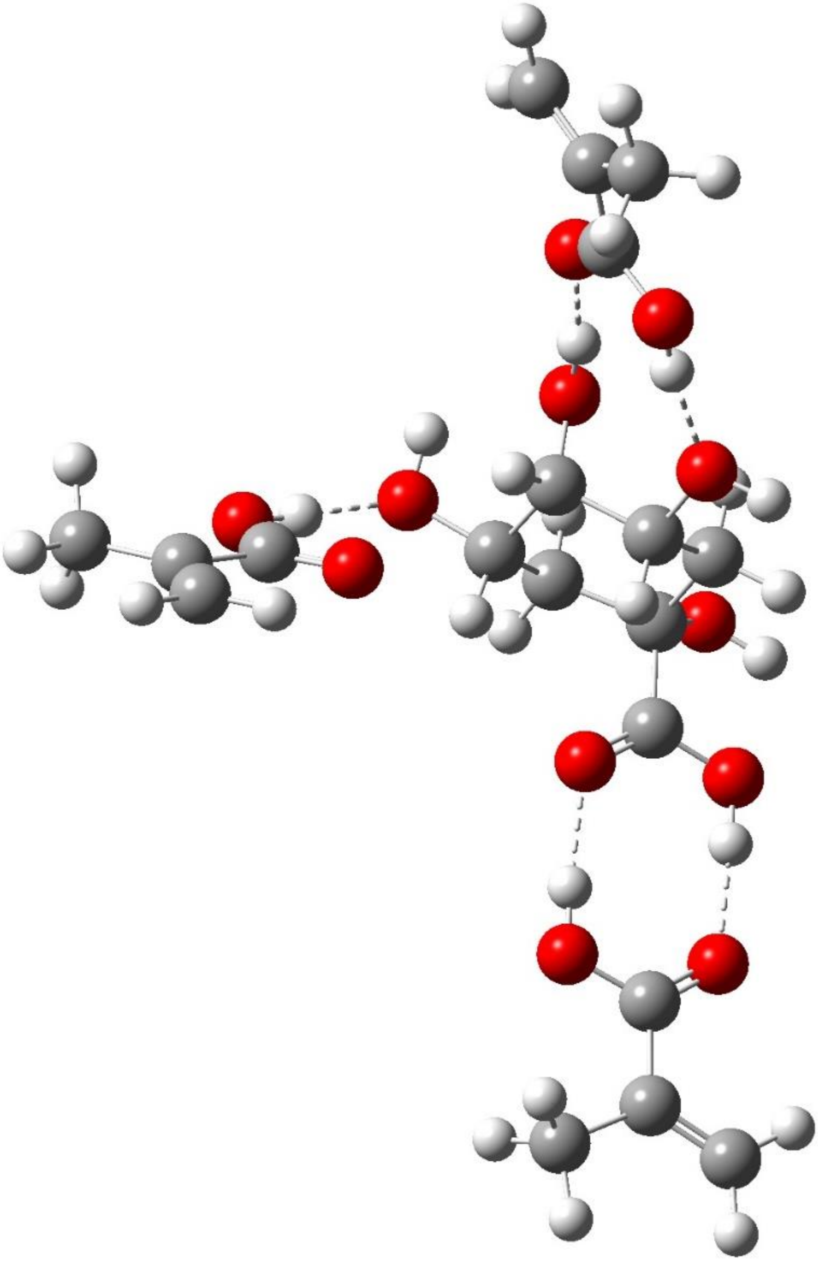

Figure S9: Computer modeled structure of the best QA-$(MAA)_3$ complex.

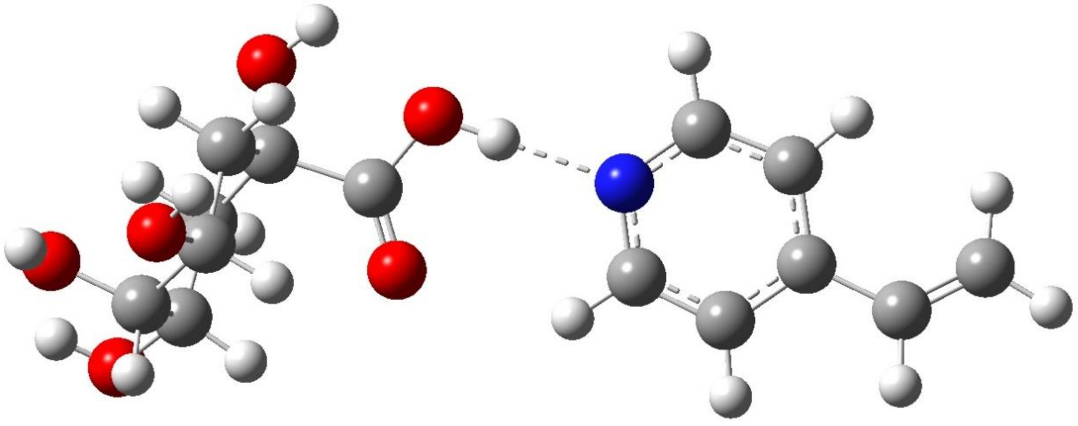

Figure S10: Computer modeled structure of the best QA-(4-VP) complex.

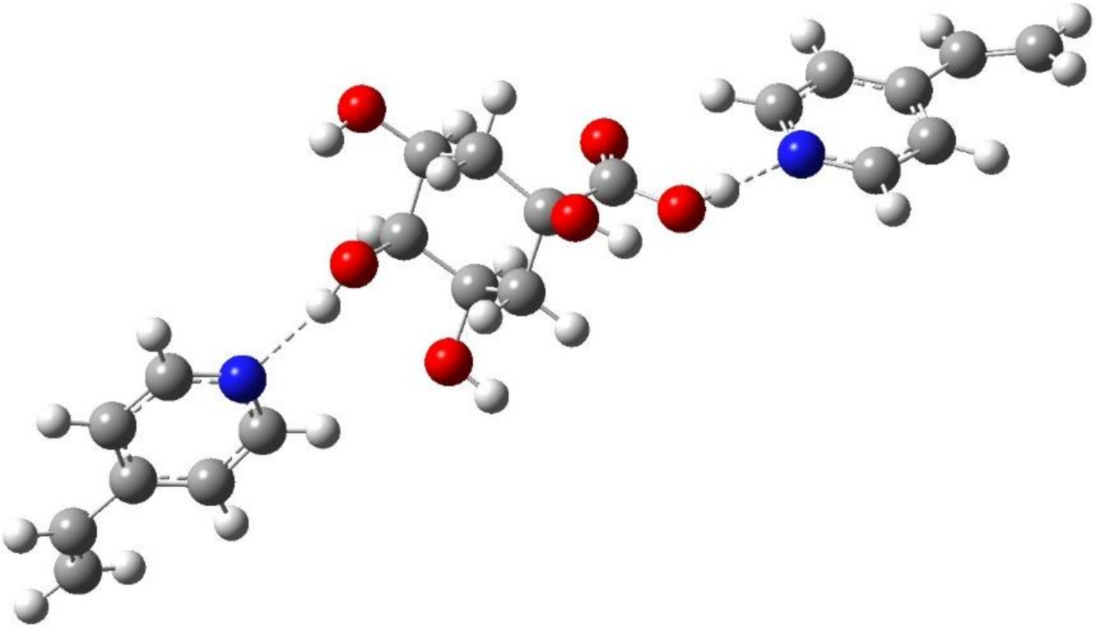

Figure S11: Computer modeled structure of the best QA-$(4\text{-VP})_2$ complex.

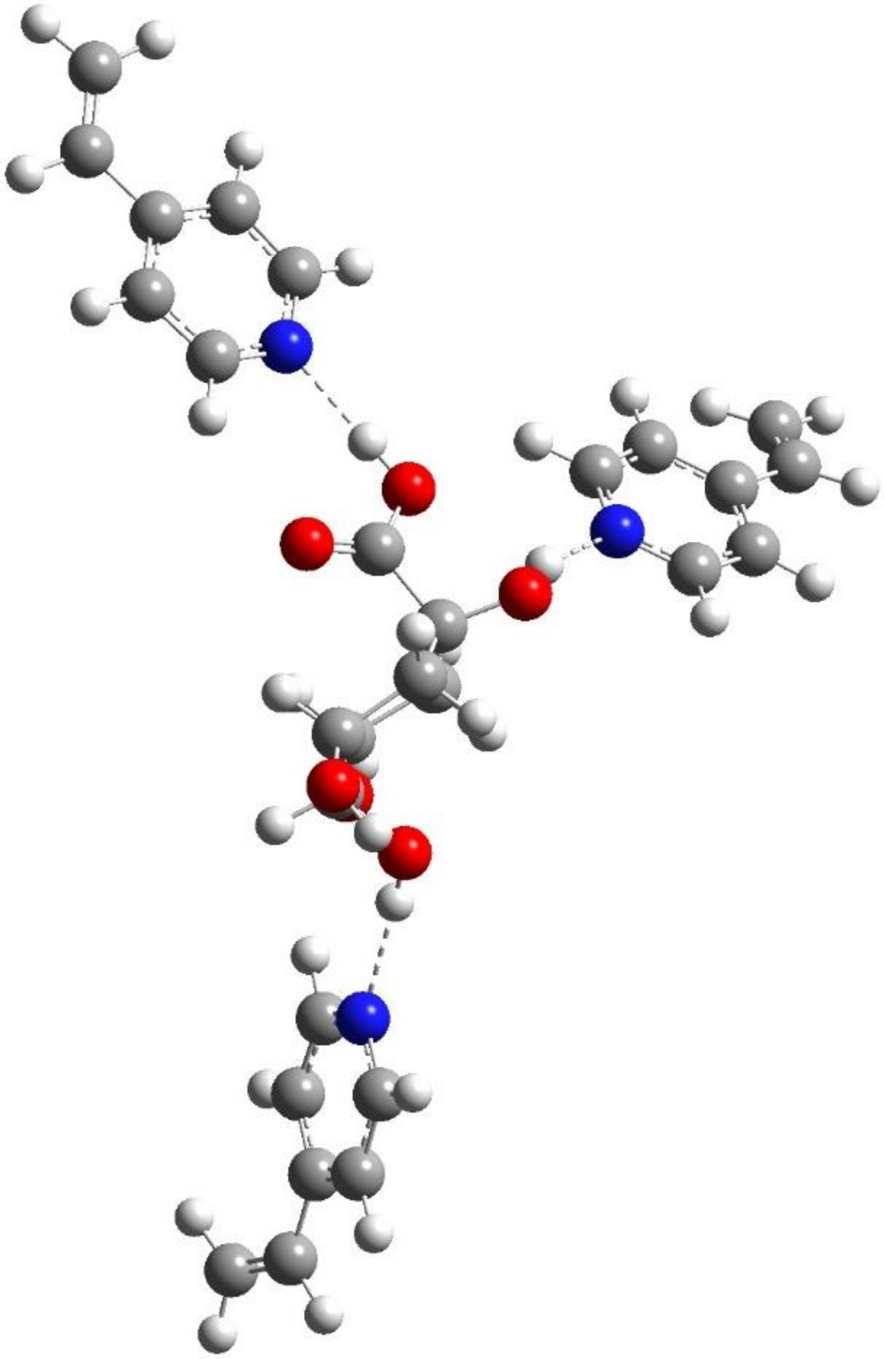

Figure S12: Computer modeled structure of the best QA-$(4\text{-}VP)_3$ complex.

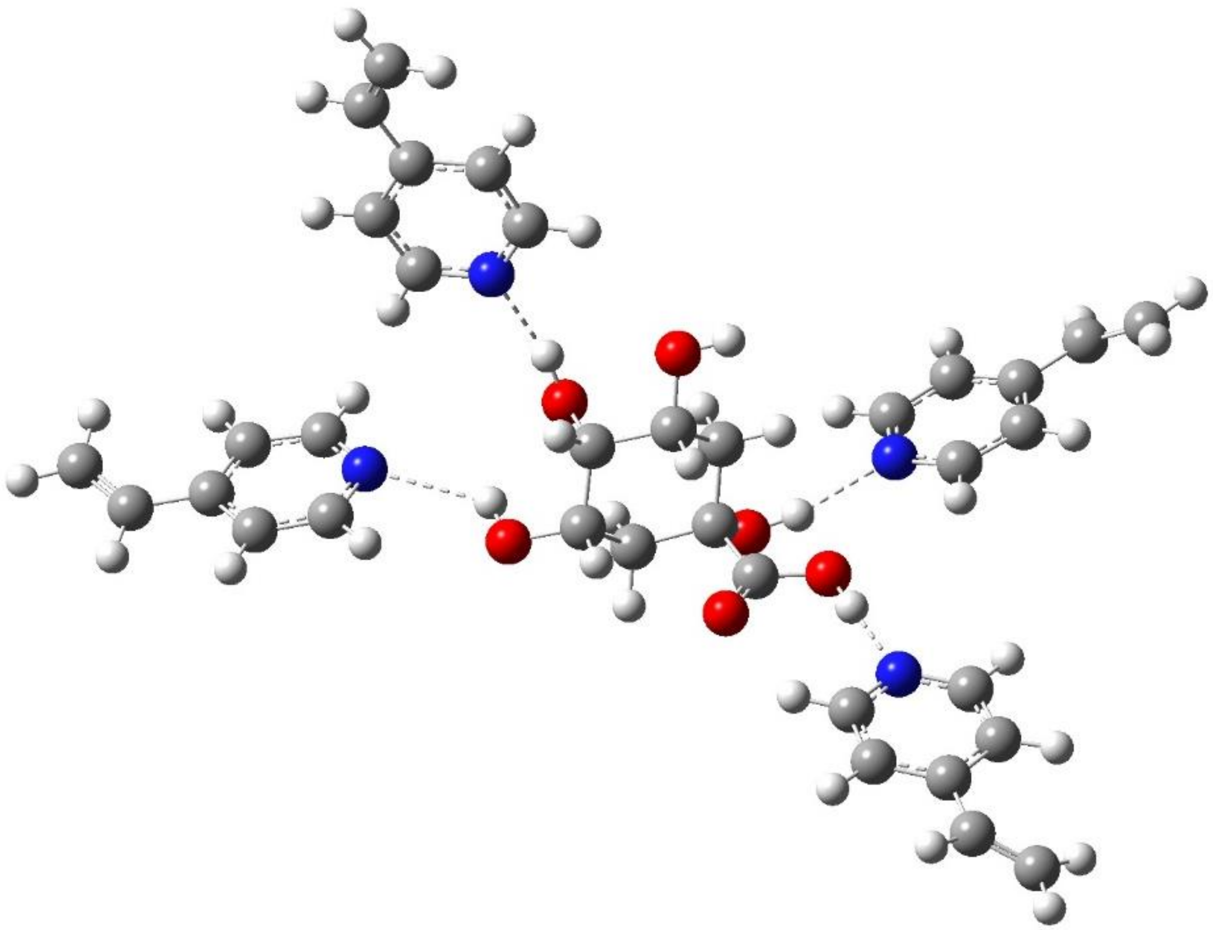

Figure S13: Computer modeled structure of the best QA-$(4\text{-VP})_4$ complex.

Table S1: The calculated binding energies for complexes prepared in solvent phase.

Allylamine Complex	ΔE KJ/mol	MAA Complex	ΔE KJ/mol	4-VP Complex	ΔE KJ/mol
**Template-(Allylamine)**	-36.757	Template-(MAA)	-401.7015	Template-(4-VP)	-49.8845
**Template-(Allylamine)$_2$**	-65.638	Template-(MAA)$_2$	-827.033	Template-(4-VP)$_2$	-73.514
**Template-(Allylamine)$_3$**	-84.016	Template-(MAA)$_3$	-1236.6105	Template-(4-VP)$_3$	-94.4885
**Template-(Allylamine)$_4$**	-110.271	Template-(MAA)$_4$	-1633.061	Template-(4-VP)$_4$	-105.02
**Template-(Allylamine)$_5$**	-154.905	-	-	Template-(4-VP)$_5$	-136.5265
**Template-(Allylamine)$_6$**	-175.909	-	-	-	-

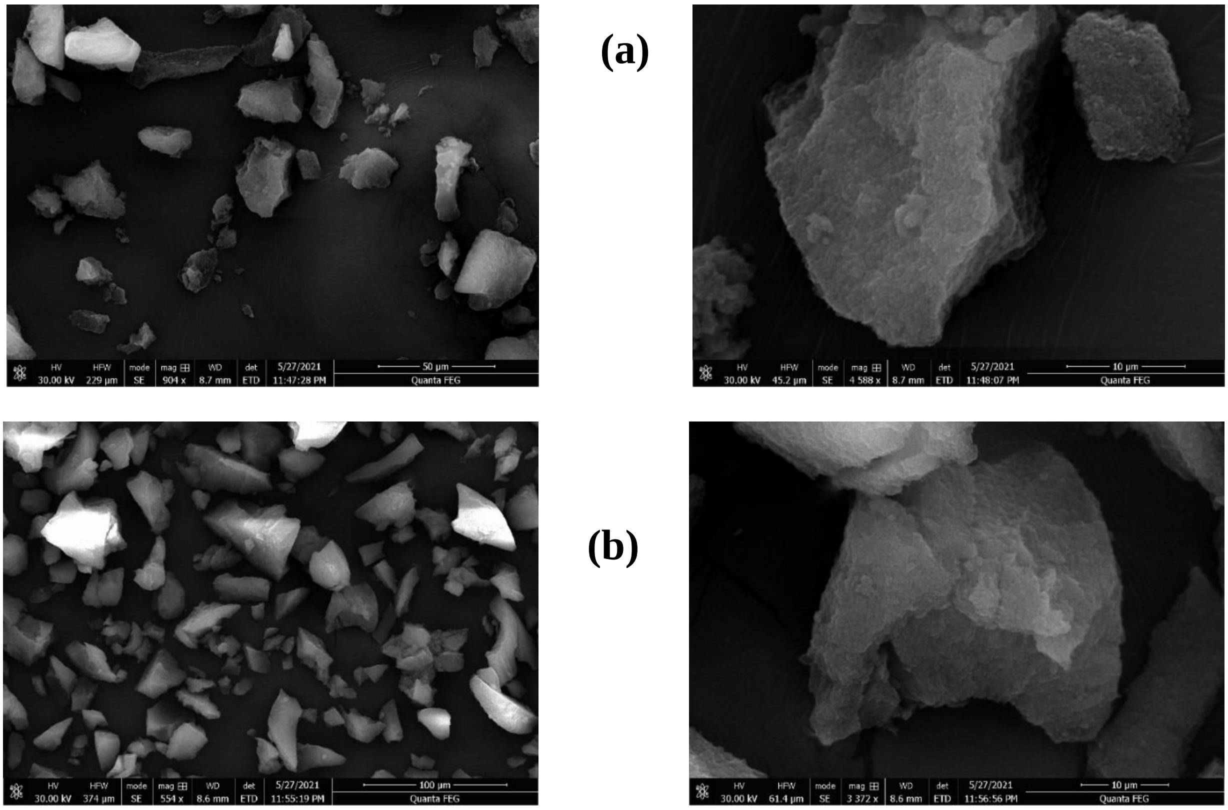

Figure S14: SEM images of (a) MIP A and (b) NIP A with increasing magnification from left to right.

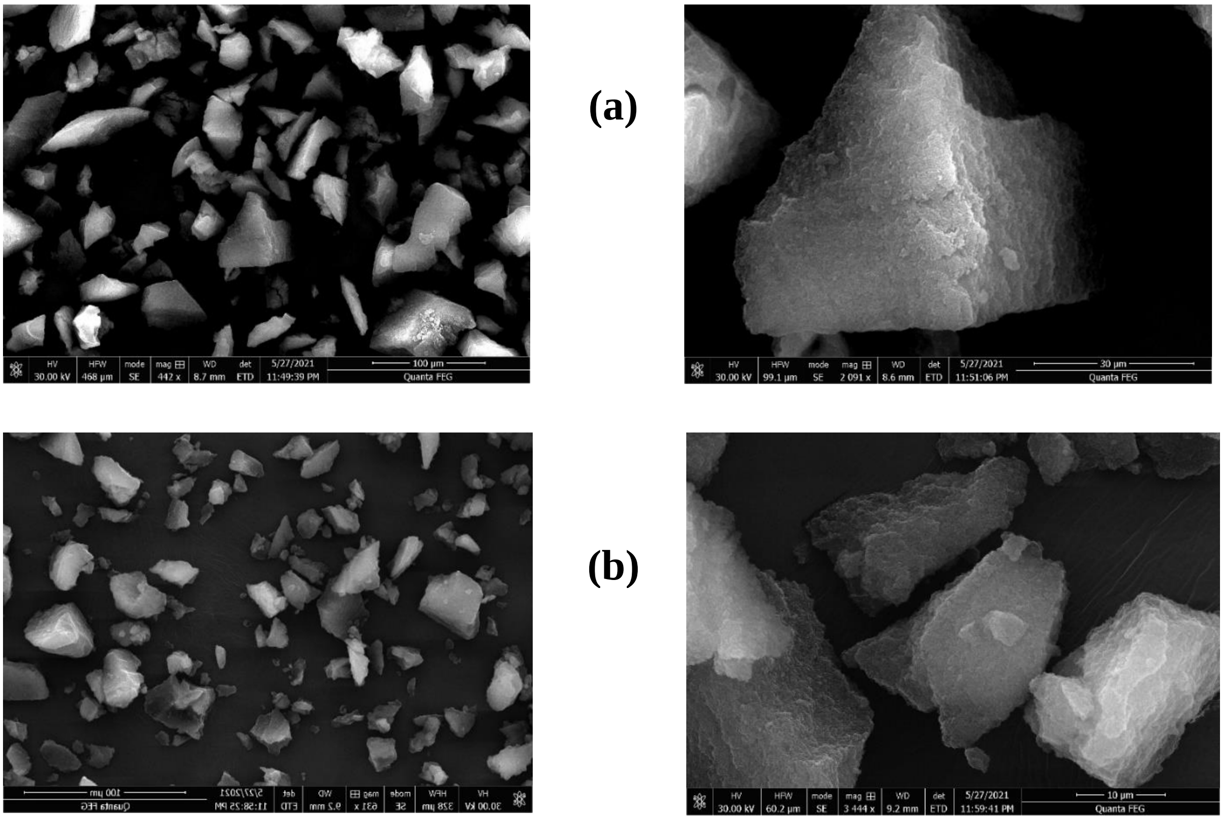

Figure S15: SEM images of (a) MIP B and (b) NIP B with increasing magnification from left to right.

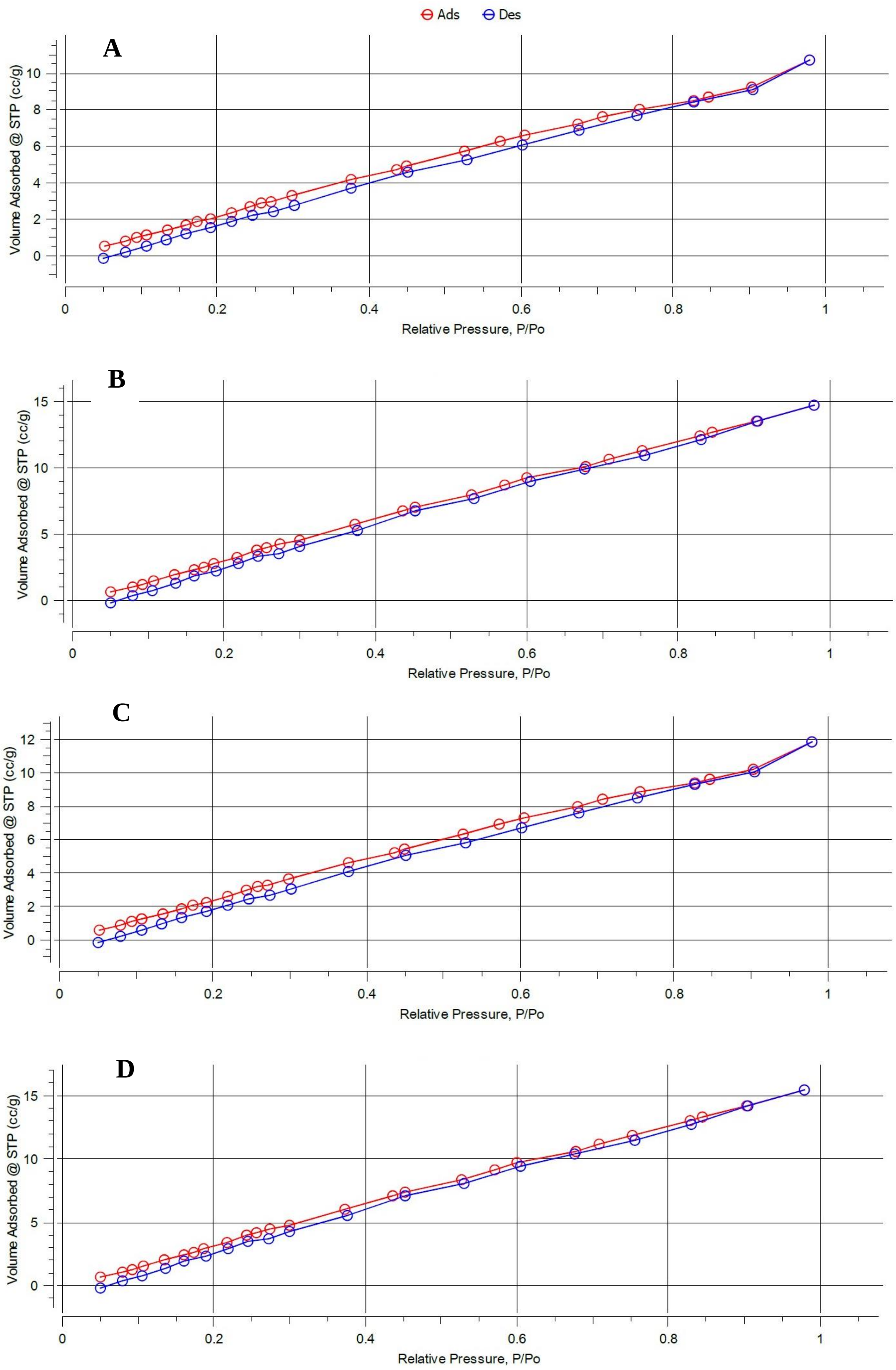

Figure S16: BET isotherms of **(A)** MIP A, **(B)** NIP A, **(C)** MIP B and **(D)** NIP B.

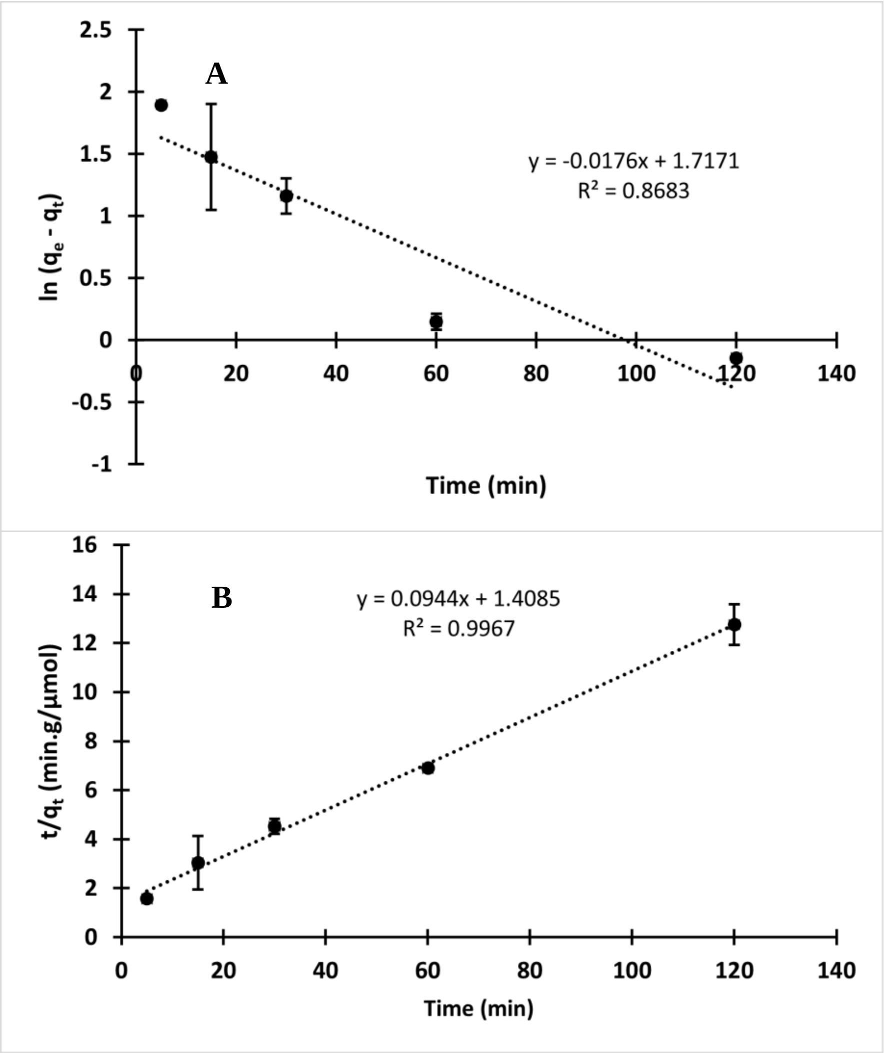

Figure S17: (A) Pseudo-first order kinetics and **(B)** pseudo-second order kinetics for MIP C.

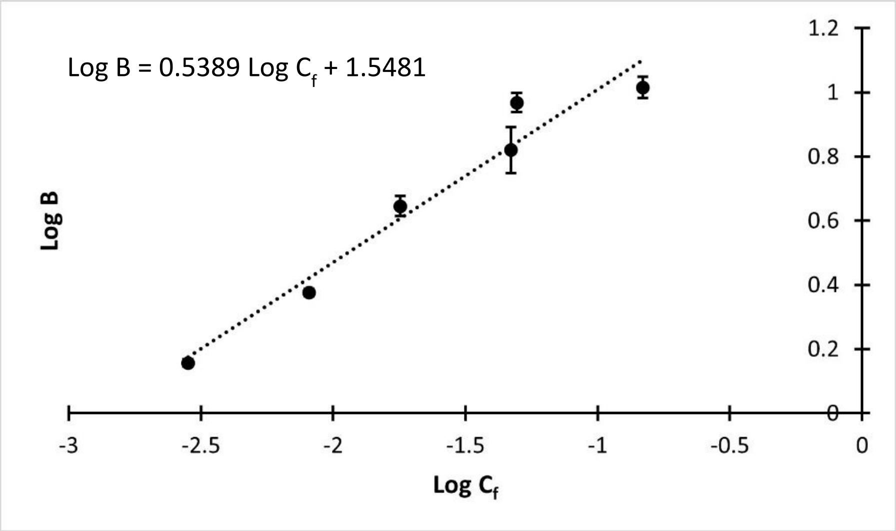

Figure S18: Freundlich isotherm for MIP C.

UHPLC-MS/MS Methods for Quinic Acid Quantification

Method Validation for QA quantification

According to the ICH guidelines, the method was validated in term of; linearity, limit of detection (LOD), limit of quantification (LOQ), precision (inter- and intra-day) and accuracy.

For linearity, an internal standard, ferulic acid with a concentration of 3 µg/ml, was used and the peak area ratio of QA/FA was plotted versus QA concentration to construct a linear calibration curve in the range of 0.001-0.2 mM made up of the seven standard solutions. Each concentration was injected 3 times. The results were evaluated by the squared regression coefficient (R^2). LOD and LOQ were calculated from the calibration curve according to **Equations (S1) and (S2).**

$$LOD = 3.3\ (\sigma/S) \qquad (\boldsymbol{S1})$$

$$LOQ = 10\ (\sigma/S) \qquad (\boldsymbol{S2})$$

where "σ" is the standard deviation of the intercept and S is the slope of the calibration curve.

Precision reflects the degree of repeatability of an analytical method. It was evaluated in this study in terms of inter- and intra-day precision which were expressed by calculating % relative standard deviation (% RSD).

Evaluation of the intra-day precision was done by analyzing three QA standard solutions (0.015, 0.03 and 0.15 mM) three times on the same day, whereas for the inter-day precision, measurements of the three samples were repeated over 3 different days.

Accuracy was evaluated by comparing the practically measured concentration by the UPLC-MS/MS instrument to the theoretical value of the three prepared concentrations of the standard. The results were expressed as recovery percentage (%R) and % RSD, based on three measurements.

The newly developed UPLC-MS/MS method was validated according to the ICH guidelines in terms of; linearity, LOD, LOQ, precision (inter- and intra-day) and accuracy. The analysis was done using 3 µg/ml ferulic acid as an internal standard, a linear calibration curve was obtained over the concentration range 0.001 – 0.2 mM. The linear correlation equation

obtained was Y = 12.639x + 0.0348 with R^2= 0.9951 **(Figure S19:** Calibration curve of QA in methanol over the concentration range of 0.001 - 0.2 mM.**Figure S19).** As shown in **Table S2**, the intra-day repeatability evaluated as % RSD ranged from 1.53 to 6.04 %, whereas for the inter-day reproducibility, the % RSD ranged from 3.84 to 8.93%. For evaluation of the accuracy, the % recovery ranged from 98.76% to 106.26% and the % RSD ranged from 4.58% to 7.21%, as shown in **Table S3**. The method has revealed low LOD (0.000265 mM) and low LOQ (0.000803 mM).

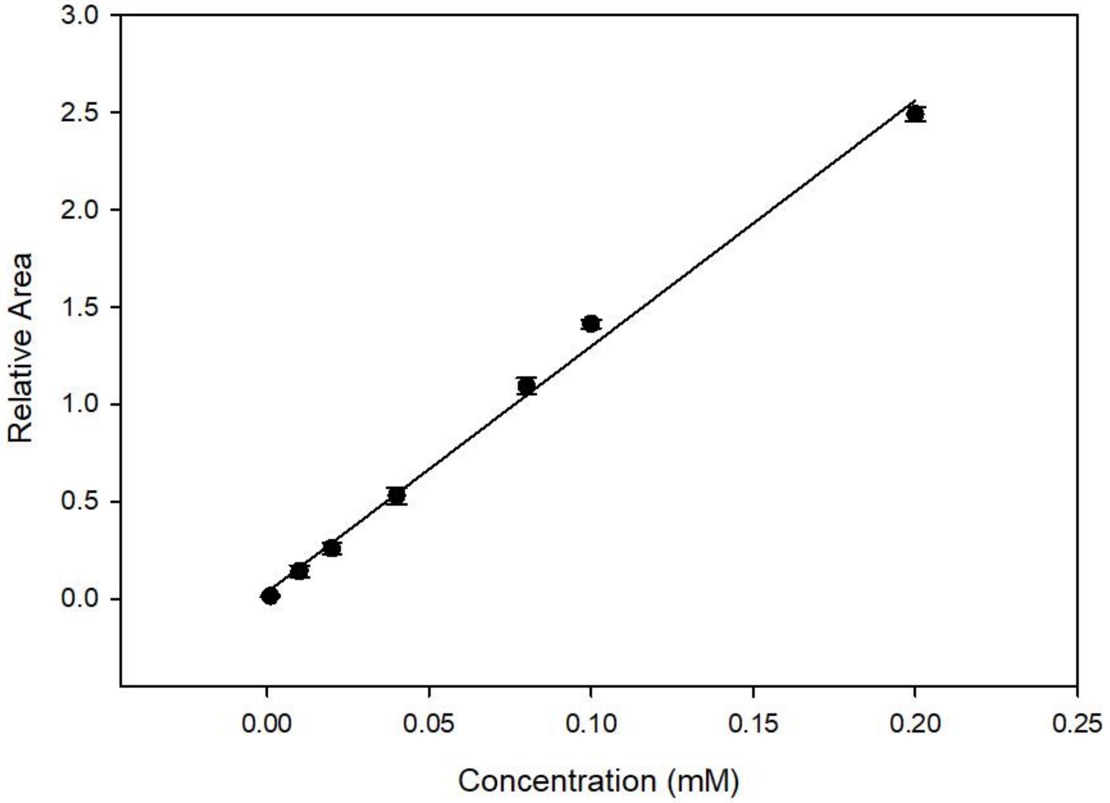

Figure S19: Calibration curve of QA in methanol over the concentration range of 0.001 - 0.2 mM.

Table S2: Intra-day and Inter-day precision of QA determination in UPLC-MS/MS method.

**Concentration (mM)**	**Intra-Day precision**		**Inter-Day precision**	
	**Peak Area Ratio**	**% RSD***	**Peak Area Ratio**	**% RSD**
**0.015**	0.21	6.04	0.23	6.93
**0.03**	0.42	1.53	0.41	3.84
**0.15**	1.90	5.03	2.05	8.93

Table S3: Accuracy of QA determination in UHPLC-MS/MS method.

**Theoretical concentration (mM)**	**Actual concentration (mM)**	**%R**	**% RSD**
**0.015**	0.0151	100.51	4.58
**0.03**	0.0296	98.76	7.21
**0.15**	0.159	106.26	5.38

Method Validation for QA quantification in coffee extract

The method was validated in term of linearity, LOD, LOQ, precision (inter- and intra-day) and accuracy. For linearity, an eight-point calibration curve was prepared in ethanol over the concentration (0.2–40 µg/ml). The results were evaluated by the squared regression coefficient (R^2). LOD and LOQ were then calculated from the calibration curve according to **Equations (S1) and (S2).**

For both intra-day and inter-day variation, standard solutions at concentrations (3, 6, and 30 µg/ml) were determined in triplicates, where intra-day variation runs where done on the same day while inter-day variation runs where done on three consecutive days. Precision was evaluated in terms of % relative standard deviation (% RSD).

For the accuracy, a recovery experiment was carried out using spiking method. 0.25 mg/ml extract was prepared and spiked with three different amounts of QA standard solutions (1x, 2x, 3x the original amounts within the extract). The extract and the spiked samples were diluted two times and were measured in triplicates and the amount of QA added was calculated by subtraction then expressed as recovery percent using **Equation (S3).**

$$\mathbf{R}\boldsymbol{ecovery}\,\% = \frac{Amount\ of\ QA\ in\ spiked\ extract -\ Amount\ of\ QA\ in\ extract\ before\ spiking}{Spiked\ amount} X100\ (\boldsymbol{S3})$$

A calibration graph for QA was constructed in ethanol using eight concentration levels injected in triplicates. A good linearity over the concentration range (0.2-40 µg/ml) was determined with square of correlation coefficient (R^2=0.9961) as shown in **Figure S20**. LOD and LOQ were calculated based on the standard deviation of the response and the slope, they were found to be 0.053 and 0.161 µg/ml, respectively.

The precision was evaluated in terms of both the percent relative standard deviations (%RSD) of the peak areas and RTs. The repeatability was tested by injecting three different concentrations (3, 6, 30 µg/ml) separately in triplicate on the same day, while inter-day precision was tested by injecting the same concentration in triplicate on three consecutive days. The %RSD obtained for peak areas were all below 5% and 7% for intra-day and inter-day assays, respectively, while %RSD for RTs ranged between 0.16 – 0.79 for intra-day assays and between 2.18 – 4.01 for inter-day assays, as shown in **Table S4**.

The method accuracy was evaluated by spiking the original extract with QA amounts~ 1x, 2x, and 3x the original concentration of QA in the extract (10, 20, 30 µg/ml). The amount of QA recovered was calculated and the percent recoveries along with %RSD were determined; the % recoveries were found to be within 96.43 - 104.81% with satisfactory %RSD, as presented in **Table S5**. Thus, the validated method showed good linearity, sensitivity, accuracy and precision.

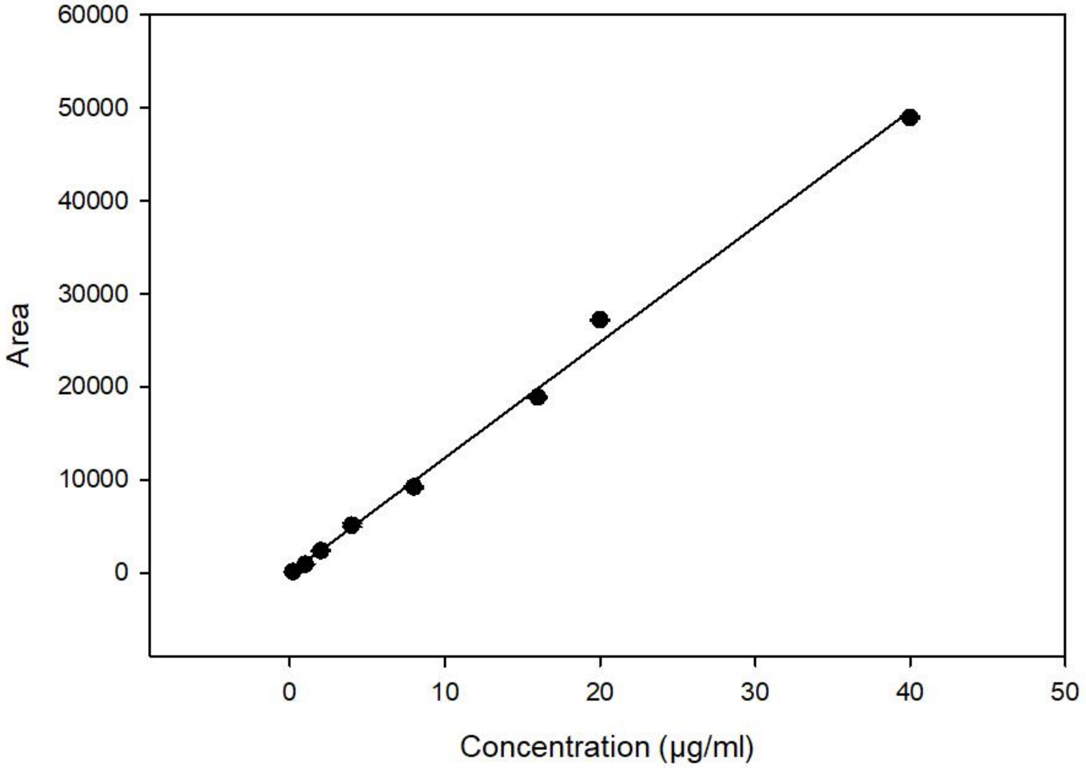

Figure S20: Calibration curve for QA in ethanol over concentration range of 0.2 - 40 µg/ml.

Table S4: %RSD of inter-day and intra-day precision assay for UHPLC measurements.

Concentration (µg/ml)	Intra-day RSD%		Inter-day RSD%	
	Peak area	RT	Peak area	RT
**3**	2.83	0.24	5.87	3.60
**6**	4.17	0.16	3.26	4.01
**30**	3.52	0.79	6.43	2.18

Table S5: Recovery % of spiked QA amount 1x, 2x, and 3x the amount of QA present in coffee extract (10, 20, and 30 µg/ml).

Amount added (µg/ml)	Amount recovered		% Recovery	
	µg/ml	%RSD	%R	%RSD
**10**	9.74	3.28	97.42	3.28
**20**	20.96	2.51	104.81	2.51
**30**	28.93	1.55	96.43	1.55
